# Tracing pesticides through terrestrial food webs with wildlife at risk

**DOI:** 10.1016/j.isci.2026.115870

**Published:** 2026-04-22

**Authors:** Shaorong Chen, Zijian Li

**Affiliations:** 1 School of Public Health (Shenzhen), Shenzhen Campus of Sun Yat-sen University, Sun Yat-sen University, Shenzhen, Guangdong 518107, China

**Keywords:** Wildlife toxicology, Environmental science, Pollution, Ecology, Ecological biochemistry, Environmental toxicology

## Abstract

Pesticides support food production but can pose ecological risks through trophic transfer in terrestrial ecosystems. Current approaches integrating contamination evidence across trophic levels with toxicokinetic and spatial models remain limited for wildlife. Here, we present an integrative framework linking empirical contamination data, physiologically based pharmacokinetic modeling, and spatial risk mapping to evaluate pesticide transfer across terrestrial food webs. Using published data from 100 studies across 30 countries, we calculated relative contamination scores for soils, plants, and wildlife and reconstructed average daily doses for representative herbivores, omnivores, and carnivores. Hazard quotients and ecological carcinogenic risks were estimated using international toxicological benchmarks. Results show pronounced spatial heterogeneity and evidence of trophic magnification for several persistent organic pollutants, with higher modeled risks in carnivores than herbivores under comparable environmental burdens. Geographic coverage remains uneven, limiting global inference. The framework is scalable but broader empirical data and independent validation are needed for robust generalizations.

## Highlights

• Wildlife risk assessment integrates pollution scoring, PBK modeling, and spatial mapping
• High-risk pesticide hotspots occur across China, India, Mexico, Brazil, and Eastern Europe
• Trophic magnification elevates pesticide risks more for carnivores than herbivores
• Foxes can act as sentinel species for global pesticide contamination

## Graphical abstract

**Figure:**
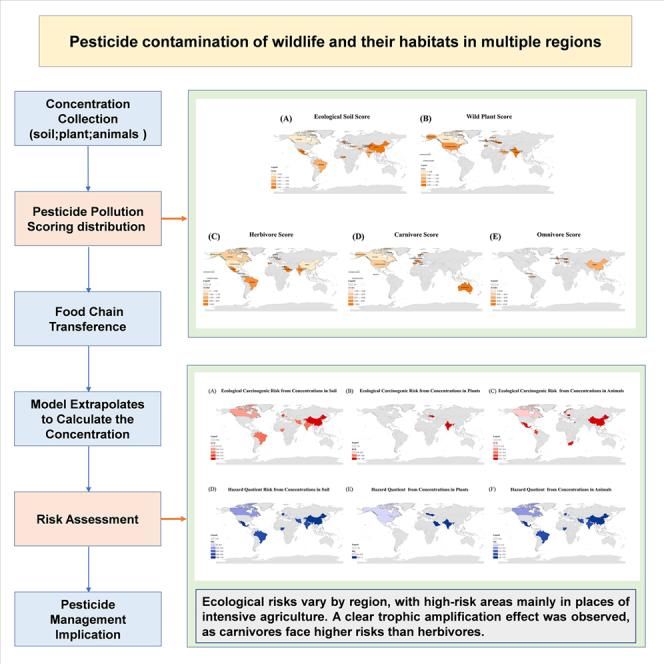

## Introduction

Pesticides are widely recognized as essential for advancing agricultural development, effectively reducing crop losses, enhancing food production and quality at reasonable cost, and contributing indispensably to global food security.^1^^,^^2^^,^^3^ By 2023, global pesticide usage exceeded 3.7 million tons annually.^4^ However, the widespread application of agricultural chemicals has led to the persistence of their residues in various environmental media.^5^^,^^6^^,^^7^^,^^8^ These residues enter non-target organisms via ingestion, inhalation, or dermal contact, adversely affecting trophic interactions and ecosystem functioning.^8^^,^^9^^,^^10^^,^^11^

The ecological risks of pesticide contamination have been recognized for decades, notably since Silent Spring highlighted the impacts of organochlorine insecticides on birds and ecosystems.^12^ Subsequent research has expanded this understanding, documenting pesticide accumulation and population-level effects across a wider range of wildlife and active ingredients.^13^ Today, pesticide contamination is understood not only as an agricultural concern but as a global ecological threat affecting wildlife and ecosystem health.^14^

Substantial progress has been made in measuring pesticide residues in specific taxa and local food webs. Bioaccumulation has been extensively studied in aquatic systems, where standardized monitoring and modeling frameworks exist. Physiologically based pharmacokinetic (PBK) models have been developed for humans and laboratory species, offering robust tools for exposure assessment. Environmental monitoring programs have also generated extensive data on pesticide occurrence in agricultural soils and crops.

Despite these advances, critical gaps remain. The transfer of pesticides from soil to terrestrial wildlife across distinct trophic levels remains poorly quantified, particularly in regions outside of intensive agricultural monitoring networks.^15^^,^^16^ The integration of bioaccumulation data across diverse species is limited, partly because transfer dynamics depend on compound-specific properties such as persistence, lipophilicity, and metabolism.^17^ Moreover, routine monitoring programs focus predominantly on agricultural areas, with limited coverage of wildlife tissues and remote ecosystems, leading to the potential underestimation of actual exposure risks. Wildlife exposure assessments are further constrained by the lack of harmonized data and toxicological linkages. PBK models, though available for laboratory species, are seldom adapted for free-ranging wildlife.^18^ As a result, standardized comparisons of species-specific exposure and associated risks across different geographical contexts remain largely speculative.

To overcome these limitations, this study establishes an integrative analytical framework that combines empirical contamination data, PBK-based exposure reconstruction, and spatial risk modeling, designed to be scalable across data-sparse environments. Unlike conventional approaches focused on single media or species, this framework traces pesticide transfer along a continuous soil-plant-wildlife continuum. By integrating empirical residue data with PBK-based internal dose modeling, this study provides a novel screening-level assessment that: (1) quantifies contamination scores across soil, plants, and wildlife from a multi-national dataset; (2) estimates pesticide exposure and calculates hazard quotients (HQs) and ecological carcinogenic risks (ECRs) using PBK and soil-plant transfer models; and (3) identifies potential hotspots of bioaccumulation and biomagnification across food chains and geographic regions.

## Results

### Global distribution of ecological pesticide contamination

**Figure 1:**
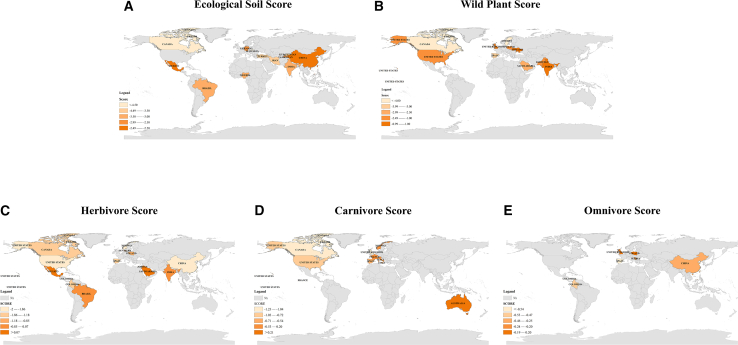
Pesticide contamination scores for soil, wild plants, and animals across countries (A) Ecological soil scores by country. (B) Wild plant scores by country. (C) Herbivore contamination scores by country. (D) Carnivore contamination scores by country. (E) Omnivore contamination scores by country. Gray areas indicate data gaps. Data are presented as raw contamination scores without aggregation; therefore, no error bars or statistical comparisons are shown.

Ecological observations across multiple trophic levels, including soils, wild plants, and faunal groups (herbivores, carnivores, and omnivores), are presented in Figure 1. Geographically, data coverage is uneven, with substantial representation from North and South America, Europe, and parts of Asia, whereas Africa and Oceania remain sparsely represented. This uneven geographic coverage (notably limited data from Africa and Oceania) constrains any global inference and should be interpreted as gaps in the literature rather than evidence of low contamination. All sampling sites are located within nature reserves or undeveloped ecosystems, minimizing local direct pesticide application, though long-range transport and adjacent agricultural use remain important pollutant sources. This ensures our findings primarily reflect ecological pollution patterns rather than direct anthropogenic inputs. The score for each trophic level characterizes internal pollution heterogeneity, with high scores indicating locally elevated pollutant loads.

For ecological soils, Mexico, China, and India rank among the top three in pollution scores, indicating significant pollutant accumulation even in protected environments. These regions are characterized by intensive agricultural practices and widespread pesticide use. In China, for instance, historical data indicate that pesticide use surged in the 1980s, coinciding with the Green Revolution’s implementation, while in India, the widespread use of chemicals such as organophosphates began in the 1970s^19^^,^^20^ In Mexico, pesticides have been heavily used in commercial farming since the mid-20th century, particularly in the production of crops such as maize and vegetables.^21^

These practices, combined with inconsistent regulation, contribute to soil degradation in conserved ecosystems via atmospheric deposition and long-range transport.^22^^,^^23^^,^^24^ However, non-agricultural sources such as industrial emissions and legacy contamination from past pesticide use in these countries also contribute to the overall pollution burden. In China, for example, industrial pollutants and mining operations have historically released heavy metals and POPs into the environment, further exacerbating the pollution problem.^25^ Given the complex interaction between agricultural practices, industrial activities, and regulatory frameworks, future studies should examine the historical patterns of pesticide use, including the classes of pesticides predominant in each region, as well as the impact of local and national regulatory policies. These drivers, along with a more detailed exploration of industrial point sources and legacy contamination, will provide a more comprehensive understanding of the regional pesticide contamination patterns.

With regard to wild plants, data from Ukraine and India indicate contamination levels that exceed the global average by 145% and 103%, respectively. The United Kingdom, the United States, and Pakistan also exceed the global average. In contrast, Canada exhibits comparatively low contamination scores, which align with its lower levels of soil contamination. The notably high scores in India highlight the strong correlation between soil and vegetation contamination. Wild plants, as primary producers directly interacting with soil, are more likely to bioaccumulate pollutants in regions with higher levels of soil contamination.^17^^,^^26^

For herbivores, Brazil, Jordan, India, and Mexico exhibited the highest pollution scores among surveyed countries, with Brazil’s levels exceeding the global average by 182%. This aligns with the elevated soil and plant scores observed in these regions. However, the United States presents an exception: Despite significant wild plant contamination, herbivore contamination scores remain relatively low. This discrepancy may stem from the broader adoption of Integrated Pest Management (IPM) strategies in U.S. agriculture, which emphasize targeted application and potentially reduce contaminant transfer to herbivores.^27^ Denmark also exhibits low herbivore contamination scores, consistent with its national policy that has nearly eliminated pesticide use on public and agricultural lands.^28^ Furthermore, spatial mismatches between sampling sites for soil, plants, and animal communities may contribute to apparent inconsistencies in observed contamination patterns.

Contamination data for carnivores and omnivores remain relatively limited. However, among carnivores, Italy and Norway exhibited the highest contamination scores among the countries studied, suggesting that pollutants may bioaccumulate through higher trophic levels. The samples in Norway were primarily foxes, a species with an elevated trophic position and higher susceptibility to biomagnification. In addition, elevated contamination levels were observed among omnivorous consumers in the United Kingdom and Spain, possibly reflecting broader dietary exposure and ecological versatility associated with omnivorous feeding habits.

These findings highlight the spatial heterogeneity and trophic-level specificity of ecological pollution within global natural ecosystems. Areas with high pollution scores often coincide with regions of intensive agricultural or industrial activity, while low-scoring temperate zones, particularly in Northern Europe and Canada, demonstrate the mitigating effects of stricter environmental controls and lower background deposition rates.

### Wildlife exposure and risk estimation

This study quantifies ecological risks to wildlife by comparing ADD of pollutants estimated from measured concentrations in environmental media and species-specific PBK models. This comparison yields two primary risk metrics: ECR and HQ (Table S26). All HQ and ECR values were Log10-transformed. For ECR, we follow commonly used risk-characterization benchmarks (ECR = 10^−6^ as negligible and ECR = 10^−4^ as high concern/unacceptable), and therefore define Log10 (ECR)>−4 as an elevated-risk category. For non-carcinogenic risk, Log10(HQ) > 0 corresponds to HQ > 1, indicating potential hazard.

**Figure 2:**
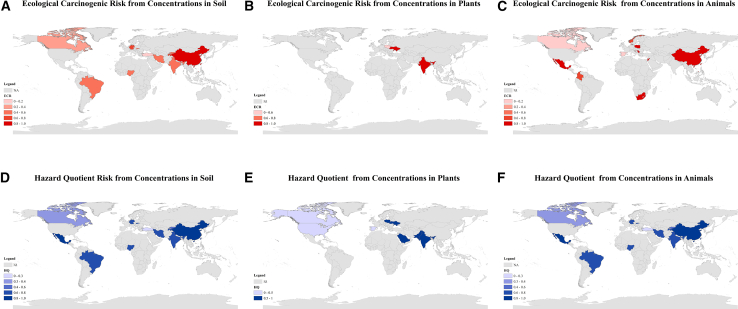
Spatial distribution of ecological carcinogenic risk (ECR) and non-carcinogenic risk (HQ) Maps display estimated ECR (A–C) and HQ (D–F) based on measured pesticide concentrations in soil (A and D), plants (B and E), and animal tissues (C and F). Risk intensities are represented by color gradients, with darker shades indicating higher risk. Higher ECR values are shown in red (A–C); higher HQ values in blue (D–F). “NA” indicates data gaps. No error bars or statistical tests are applicable to these spatial maps.

**Figure 3:**
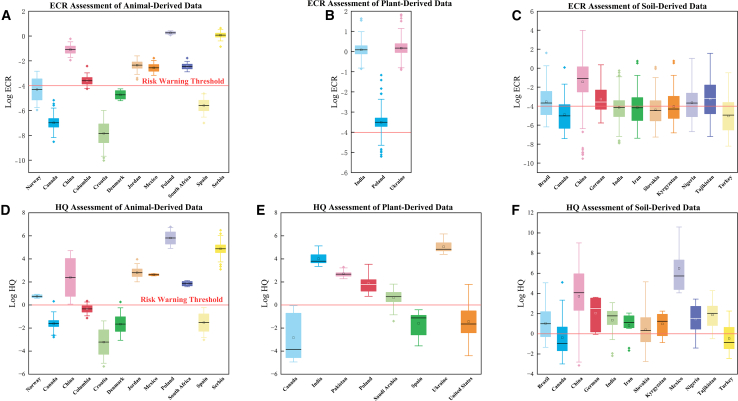
Transnational risk assessment of ecological carcinogenicity (ECR) and non-carcinogenicity (HQ) based on pesticide concentrations in soil, plants, and animals (A–C) show ECR for animal, plant, and soil data, respectively, with values on a logarithmic scale (Log ECR) and the risk warning threshold indicated by the red line. D-F -6display HQ values for animal, plant, and soil data, also on a logarithmic scale (log HQ). The red line indicates the risk warning threshold (log ECR = 10–4 in A–C; log HQ = 0 in D–F).

Across the compiled dataset, HQ and ECR estimates exhibited substantial spatial heterogeneity among surveyed countries (Figures 2 and 3). For Log10 (HQ), soil-derived estimates more frequently exceeded 0 than plant- or tissue-derived estimates, suggesting that soil can represent an important exposure driver in many sampled settings. Within the available dataset, samples from China, India, Brazil, and Mexico showed relatively higher Log10 (HQ) values (>0) compared with other surveyed countries, including samples collected from protected areas (Figure 3F). These patterns appear as localized areas of elevated relative estimates in the spatial visualization (Figure 2D); however, the map reflects data availability and should not be interpreted as a definitive global hotspot ranking. Elevated HQ values derived from plant concentrations in India, Ukraine, Poland, and Pakistan further suggest that vegetation can represent an important uptake and exposure pathway in some regions (Figure 3E). In animal tissues, HQ estimates were generally lower and more heterogeneous, but relatively higher values observed in Brazil, Mexico, Poland, and Serbia are consistent with the possibility that trophic transfer and bioaccumulation contribute to cumulative exposure in some food webs (Figure 3D).

Ecological carcinogenic risk patterns (expressed as logarithmic ECR) exhibit similar yet more concentrated spatial heterogeneity (Figure 2). Soil-mediated Log10 (ECR) values above −4 were observed in samples from China, Mexico, Tajikistan, and Germany (Figure 3A), indicating elevated risk estimates within the compiled dataset. For plant-based exposure, detectable ECR values above the benchmark were observed in India, Poland, Pakistan, and Ukraine (Figure 3B). Elevated ECR estimates in animal samples (e.g., Serbia, Poland, Mexico, and China) are consistent with higher-trophic-level accumulation reported in previous work, although the limited number of carnivore and omnivore studies constrains inference.^18^

**Figure 4:**
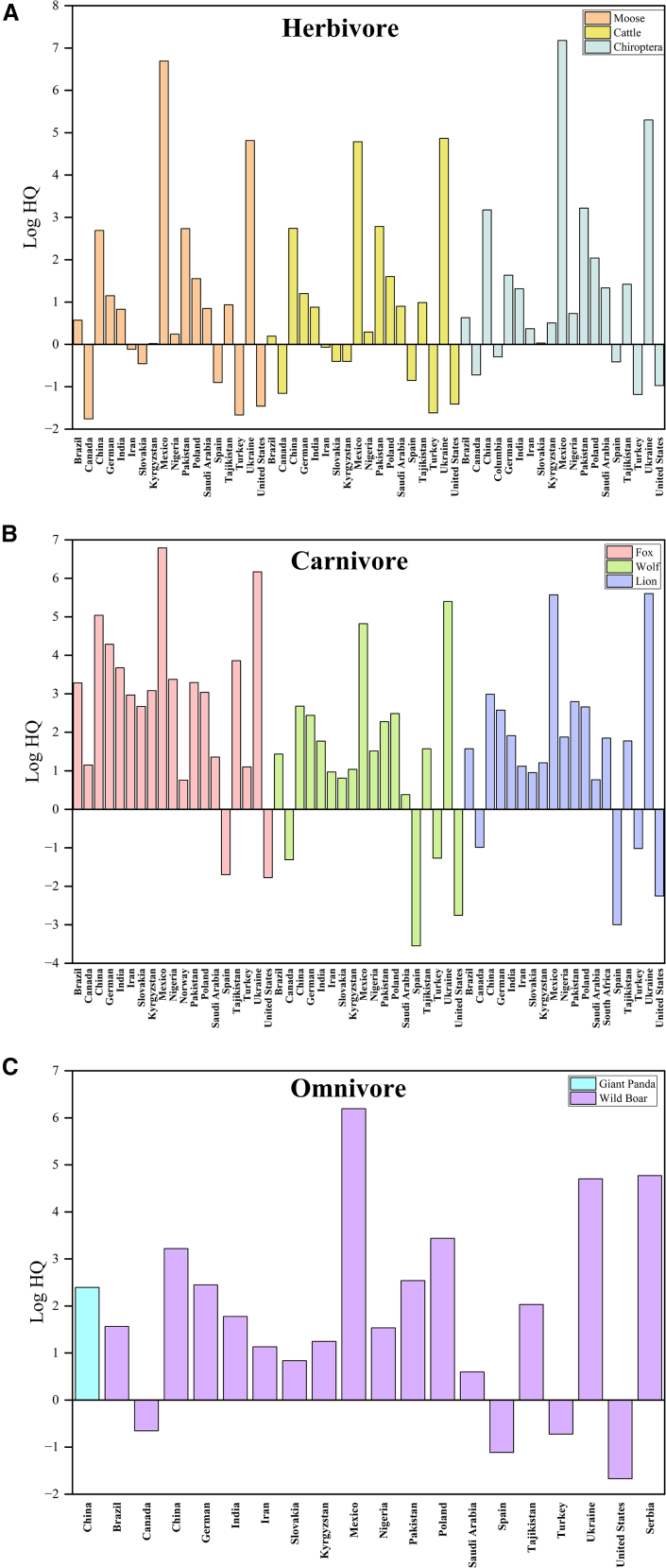
Non-carcinogenic risk (log HQ) for species with different diets across surveyed countries Bars represent the mean log HQ value for each dietary group (herbivores, carnivores, and omnivores). No error bars or statistical comparisons are shown, as the figure is intended for descriptive visualization of point estimates. A log HQ value greater than zero indicates a risk that requires consideration.

To further elucidate species-specific exposure patterns, risk assessments based on the ADD approach were conducted for representative populations (Figure 4). Herbivores in Poland, China, Pakistan, Mexico, and Ukraine had the highest non-carcinogenic risks (Figure 4), reflecting regional differences in agricultural practices and regulation.

For carnivores (such as foxes, wolves, and lions), the risk patterns are more complex. Elevated non-carcinogenic headquarters counts were recorded in Germany, China, Ukraine, and Mexico. HQ exhibits significant interspecies variation: Foxes demonstrate non-carcinogenic potential in most surveyed regions except Spain and the United States, while wolves and lions show negligible non-carcinogenic risk in Spain, the United States, Canada, and Turkey. This indicates that species-specific nutritional niches, metabolism, and prey specialization significantly modulate exposure dynamics.

Among omnivorous animals (such as wild boars and giant pandas), both regional and species-dependent variations are highly pronounced. Wild boars in Mexico exhibited the highest non-carcinogenic risk, followed by those in Serbia, Ukraine, and China. In contrast, samples from Spain, the United States, Canada, and Turkey showed no non-carcinogenic risk. For giant pandas, data are limited to China, indicating measurable non-carcinogenic hazards, but insufficient comparative evidence exists for broader spatial extrapolation.

## Discussion

### Pesticide transport through ecological food chains

Six representative pesticides were selected from the 34 compounds analyzed, as illustrated in the full list provided in the supplemental dataset. As demonstrated in Table 1, the pollution score analysis revealed substantial disparities in pollutant loads across various ecological trophic levels. The findings indicate that carnivores exhibit markedly higher median values for Log ECR and Log HQ compared to herbivores, reflecting the transfer and bioaccumulation of pollutants along the food chain from primary producers to higher trophic level consumers. This finding is consistent with the established bioaccumulation patterns documented in previous studies.^29^^,^^30^ Within the herbivore group, bats exhibited substantially elevated risk levels compared to larger species such as deer and cattle; similarly, among carnivores, foxes showed higher risk levels than wolves and lions (Table 1). This pattern suggests that smaller-bodied species face elevated risks under equivalent environmental concentrations, partly explained by higher exposure per unit body mass.^31^^,^^32^ In the PBK model used here, body weight (BW) is indeed a key parameter influencing pollutant accumulation and metabolism. However, ecological traits such as foraging behavior, dietary breadth, and habitat use—exemplified by bats’ mobility and foxes’ omnivores—further modulate exposure. Incorporating these ecological dimensions into physiological modeling improves understanding of wildlife pesticide exposure.

**Table 1:** Risk assessment of six common pesticides at different trophic levels

	Aldrin		HCB		p,p'-DDT		Heptachlor		Chlorpyrifos		Imidacloprid	
	Log ECR	Log HQ	Log ECR	Log HQ	Log ECR	Log HQ	Log ECR	Log HQ	Log ECR	Log HQ	Log ECR	Log HQ
Caribou	−5.13	**0.82**	−6.75	−2.21	−5.02	**0.4**	−4.89	−0.60	__^a^	**1.35**	__	−1.01
Cattle	−5.26	**0.98**	−7.16	−2.32	−5.02	**0.69**	−5.02	−0.43	__	**1.51**	__	−0.85
Chiroptera	**−2.93**	**1.41**	−4.84	−1.89	**−2.95**	**0.88**	**−2.7**	**0.002**	__	**1.95**	__	−0.41
Moose	−5.34	**0.93**	−7.25	−2.38	−5.35	**0.39**	−5.11	−0.48	__	**1.46**	__	−0.90
Rabbit	−4.64	**0.50**	−6.54	−2.80	−4.53	**0.09**	−4.40	−0.91	__	**1.04**	__	−1.33
Sheep	−5.07	**0.70**	−6.70	−2.33	−4.84	**0.41**	−4.76	−0.64	__	**1.23**	__	−1.13
Fox	**−1.18**	**4.04**	**−3.28**	**0.55**	**−1.29**	**3.41**	**−1.01**	**2.57**	__	**3.29**	__	−3.02
Wolf	**−3.71**	**2.12**	−6.15	−1.79	**−3.62**	**1.68**	**−3.68**	**0.50**	__	**2.28**	__	−3.97
Lion	**−3.71**	**2.26**	−6.10	−1.53	**−3.38**	**2.04**	**−3.67**	**0.65**	__	**2.79**	__	−3.42
Wild boar	**−3.64**	**2.20**	−5.73	−1.28	**−3.42**	**1.91**	**−3.39**	**0.81**	__	**2.54**	__	−1.54

Median values of log ecological carcinogenic risk (ECR) and log hazard quotient (HQ) for various species. A Log ECR greater than 4 indicates a significant carcinogenic risk, while a Log HQ greater than 0 indicates a potential non-carcinogenic risk. Bolded annotations in the table indicate the presence of risk.

a Indicates no corresponding data results.

The selected chemicals include both historically used persistent organic pollutants (POPs) and modern, commonly used pesticides. POPs, such as aldrin and p,p′-DDT, exhibit high log octanol-water partition coefficients and long environmental half-lives, which hinder biotransformation and favor lipid storage. As shown in Table 1, foxes displayed substantially higher Log HQ values for aldrin (4.04) and p,p′-DDT (3.41) compared to typical herbivores such as cattle (aldrin = 0.98; p,p′-DDT = 0.69), confirming a strong trophic magnification pattern. The estimated biotransformation factors (BTF>1) further support the persistence-driven bioaccumulation of these legacy compounds through terrestrial food webs. In contrast, modern non-persistent pesticides such as chlorpyrifos and imidacloprid show divergent patterns. Chlorpyrifos, despite its relatively short environmental persistence, exhibits high acute toxicity and a measurable potential for secondary poisoning, with Log HQ > 0 even in herbivores, and further elevated values in carnivores.^33^ Conversely, imidacloprid exhibited consistently negative Log HQ values across all trophic levels, indicating no trophic magnification within the studied ecosystem. However, this finding should be interpreted with caution, as our tissue-based approach does not capture imidacloprid’s well-documented acute toxicity to invertebrates or its indirect effects on higher predators via prey depletion.^34^^,^^35^

Integrating spatial pollution scores with species-specific risk profiles reveals that food web-mediated exposure is most pronounced in regions such as China, India, and Mexico, where intensive agriculture and unregulated pesticide use lead to elevated soil and plant burdens that cascade upward through food webs.^36^^,^^37^ Within these ecosystems, foxes may experience relatively higher exposure due to their upper trophic position, suggesting potential value as sentinel candidates.^38^ Conversely, lower trophic transfer intensities were observed in North America and Western Europe, where restrictions on legacy POPs and adoption of integrated pest management have reduced baseline burdens.

### Uncertainty and interpretation of estimated ecological health risks

**Figure 5:**
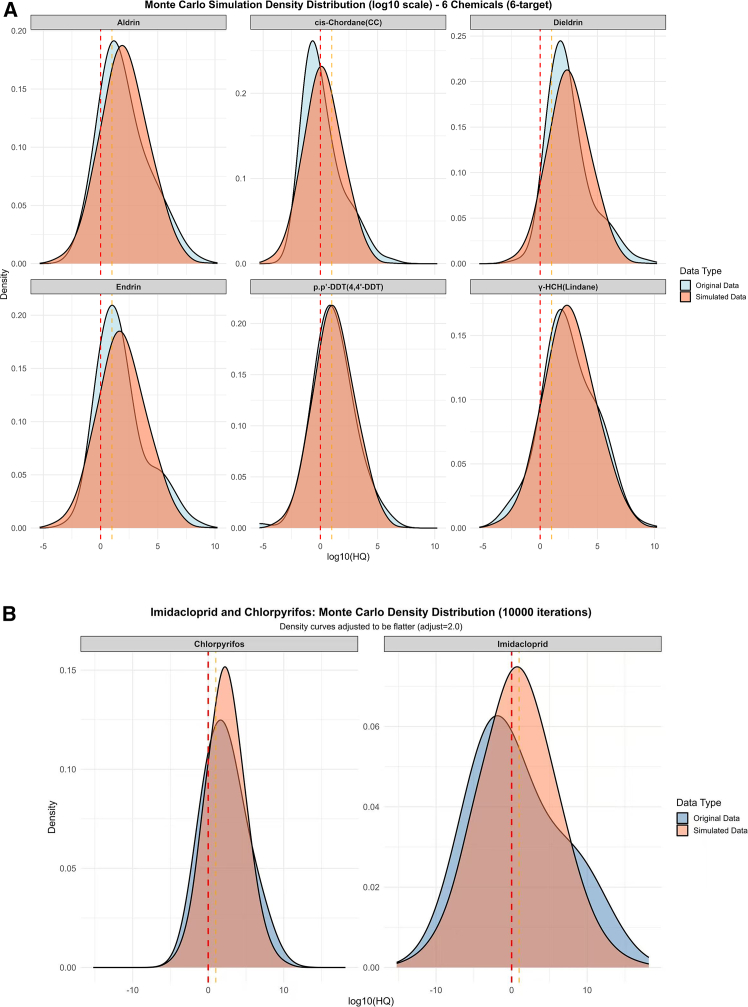
Results of Monte Carlo simulations with 10,000 iterations for six persistent organic pollutants (POPs) and two modern pesticides (Chlorpyrifos and Imidacloprid) Blue curves represent the distribution of original observed data; orange curves represent simulated data. Probability density is shown on the y axis. No error bars or statistical tests are presented for this figure.

To further assess the uncertainty and variability in HQ estimates, Monte Carlo simulations (10,000 iterations) were conducted for representative POPs and modern pesticides. Figure 5 presents the simulated probability density distributions (Log-HQ scale) compared with observed data across chemical categories.

As shown in Figure 5A, the six POPs (Aldrin, cis-Chlordane, Dieldrin, Endrin, p,p′-DDT, and γ-HCH) exhibited approximately log-normal HQ distributions, with simulated curves closely matching empirical data, indicating robust model fit. The mean simulated HQ values generally exceeded unity (Log HQ > 0) for Aldrin, Dieldrin, and p,p′-DDT, suggesting a high probability of ecological risk persistence under realistic exposure conditions.

The right-skewed tails for Aldrin and Dieldrin indicate scenarios where HQ could exceed thresholds by several orders of magnitude, reflecting their strong bioaccumulation potential. In contrast, γ-HCH and cis-Chlordane displayed narrower distributions, implying lower variability and reduced bioaccumulation potential. The overall pattern aligns with their physicochemical properties, specifically high Log K_OW_ (>5) and long half-lives, that promote persistence and magnification in upper trophic levels. These results confirm the trophic amplification patterns observed above and underscore the difficulty of mitigating legacy pesticide residues in natural ecosystems.

Monte Carlo density simulations have been conducted for Chlorpyrifos (an organophosphate) and Imidacloprid (a neonicotinoid) in Figure 5B. Chlorpyrifos shows a relatively compact, symmetrical distribution with a median close to 3, indicating moderate but consistent non-carcinogenic risk under varying environmental exposure levels. This indicates that despite its lower persistence than POPs, its acute toxicity may pose localized risks, especially in intensive agricultural regions. Conversely, imidacloprid displays a more extensive, right-skewed distribution with a central tendency below zero (Log HQ < 0), indicating minimal non-carcinogenic risk under most simulated conditions. The observed skewness indicates that, in certain high-exposure scenarios (e.g., direct plant uptake or pollinator ingestion), the compound may transiently reach risk-relevant concentrations. This variability is indicative of the unique exposure dynamics of systemic pesticides; their risk is contingent on insect-pesticide interactions as opposed to classical trophic accumulation processes.

**Figure 6:**
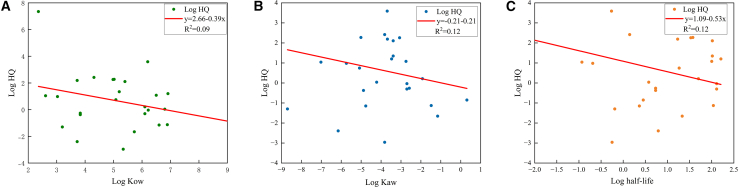
The correlation and trends between the log HQ value and the physicochemical properties of chemical substances (A) Log HQ vs. log Kow (octanol-water partition coefficient). (B) Log HQ vs. log Kaw (air-water partition coefficient). (C) Log HQ vs. log half-life (biotransformation half-life). Points represent individual pesticides. The solid lines show the linear regression fits, and the coefficient of determination (R^2^) is reported for each regression.

The relationship between Log HQ and three key physicochemical parameters—octanol-water partition coefficient (Log Kow), air-water partition coefficient (Log Kaw), and biotransformation half-life (Log half-life)—is shown in Figure 6. A weak negative correlation exists between Log HQ and Log Kow, as well as Log half-life (Figures 6A and 6C). Although Log Kow and half-life are standard indicators of bioaccumulation potential, our study shows that high values do not necessarily correspond to elevated HQ in wildlife risk modeling. This observed trend suggests that the current risk (as measured by HQ) is not solely determined by these inherent properties but is more significantly influenced by actual environmental exposure levels. Several factors explain this divergence. First, many highly lipophilic and persistent compounds (e.g., DDT, dieldrin, and endosulfan) have been banned or severely restricted under the Stockholm Convention, leading to a significant reduction in their environmental concentrations despite their high bioaccumulation potential.^39^ Second, pesticides with lower Kow values and faster degradation rates (e.g., organophosphates and neonicotinoids) are currently more widely used, resulting in higher environmental residues and consequently elevated HQ values, even though their intrinsic bioaccumulation capacity is lower.^37^^,^^40^ These findings suggest that, in the case of current ecological risk, the intensity of exposure, driven by usage patterns, outweighs the chemicals’ inherent hydrophobicity and persistence in the environment. This observation aligns with the hypothesis that recent pesticide applications are the primary cause of the majority of wildlife toxicity incidents.^41^^,^^42^^,^^43^

Log HQ values also demonstrate a weak negative correlation with logarithmic Kaw values (Figure 6B), indicating that compounds with lower volatility (smaller Kaw values) tend to exhibit higher HQ values. This pattern likely arises because less volatile compounds persist in soil and vegetation, increasing transfer to herbivores and higher-order consumers. In contrast, highly volatile compounds partition more readily into the atmosphere, undergoing rapid dilution and degradation, which results in lower local exposure concentrations.

In the sensitivity analysis conducted on the ECR model for fox liver concentrations, the Sobol indices revealed key insights into the factors driving uncertainty in the model output (Figure S1). The total-effect Sobol index (ST) for BW was the highest at 0.505 (95% CI: 0.459–0.553), indicating that BW alone explains approximately 50% of the variance in the ECR output. The first-order Sobol index (S1) for BW was 0.380 (95% CI: 0.299–0.450), suggesting that BW’s direct effect is the dominant contributor to model uncertainty, with a notable interaction effect (ST − S1 = 0.125). The oral slope factor (OSF) also emerged as a significant contributor to uncertainty in the model (ST = 0.441, 95% CI: 0.398–0.485; S1 = 0.302, 95% CI: 0.256–0.341), accounting for approximately 44% of the total variance. The interaction between OSF and other parameters was also relatively strong (ST−S1 = 0.139), indicating a considerable level of dependence between OSF and other parameters, especially in terms of the toxicokinetic and toxicodynamic processes. Liver Tissue Concentration (C_tissue_), representing the exposure input for the model, demonstrated the lowest contribution to model uncertainty. The total-effect index for C_tissue_ was 0.070 (95% CI: 0.042–0.098), with a first-order index of 0.035 (95% CI: 0.024–0.046). These findings suggest that despite being a key input in the model, the variability in C_tissue_ has a relatively minor impact on the uncertainty of the ECR output, potentially due to its narrow range of variability and lower model sensitivity compared to BW and OSF.

In addition to the direct sensitivity analysis for the fox liver concentrations, a Sobol sensitivity analysis was conducted for the ECR model based on soil data, (Figure S2). The most significant parameter was Fox’s food intake (IR_fox_), with an ST of 0.745 (95% CI: 0.689–0.833), accounting for a large portion of the variance in the ECR model. The S1 for IR_fox_ was 0.351 (95% CI: 0.174–0.472), showing its dominant direct effect. BTF_fat, rabbit_ and BTF_muscle, rabbit_ also contributed notably, with total-effect indices of 0.295 and 0.286, respectively, and first-order indices of 0.040 and 0.049. These factors had smaller effects than IR_fox_, but were still important. BW_fox_ showed a total-effect index of 0.218 and a first-order index of 0.035, indicating a secondary role in the model. In contrast, IR_rabbit_, BAF_plant_, and C_soil_ had minimal impacts, with Csoil showing a negative total-effect index of −0.0007, suggesting an inverse or negligible influence on the ECR.

### Implications for pesticide management and policy

Field observations of wildlife poisoning provide contextual support for the modeled risk patterns. Raptor mortality linked to organophosphates and carbamates has been documented across Europe, Asia, and Africa.^44^^,^^45^^,^^46^ In India, vulture population declines have been attributed to secondary poisoning from diclofenac and organochlorines.^47^ Pesticide-induced mortalities have also been reported in foxes, wild dogs, and badgers.^42^ The United Nations Environment Program has identified immunotoxicity in animals for all ten banned persistent pesticides, with some compounds also showing carcinogenicity.^48^^,^^49^^,^^50^^,^^51^ These documented effects align with regions where our modeling indicated elevated ecological risk (e.g., India, China, Mexico, and parts of Eastern Europe), reinforcing the relevance of integrating wildlife exposure data into pesticide management frameworks.

By integrating PBK modeling with empirical residue data in a multi-trophic framework, this study offers a more holistic representation of real-world exposure. Although agencies such as EFSA and USEPA have begun exploring multi-trophic modeling in risk assessment, implementation remains fragmented.^52^^,^^53^ In the future, pesticide management should replace or supplement human health benchmarks (RfD, OSF) with ecologically relevant thresholds for key wildlife species.

Furthermore, pharmacokinetic (PBK)-based bioaccumulation and risk assessments should be required for pesticides with log Kow >4 or for which there is evidence of trophic transfer. In addition, systematic monitoring programs for non-target wildlife should be established for high-risk areas identified using spatially explicit models.

### Limitations of the study

We acknowledge that using human health-based toxicity benchmarks (USEPA RfD and OSF) for wildlife risk assessment introduces uncertainties related to physiological differences, species sensitivity, and protection goals. These benchmarks are therefore used as a preliminary screening tool, pending the development of species-sensitive ecological thresholds by agencies such as EFSA.

Extrapolating PBK parameters and BTFs from laboratory or domesticated species to free-ranging wildlife introduces uncertainty. Although allometric scaling and species-specific data were applied where possible, unmeasured interspecific differences in metabolism, tissue partitioning, and elimination may bias predictions. Sensitivity analyses identified BW and intake rates as key drivers of uncertainty, highlighting the need for improved species-specific inputs and independent validation.

To estimate pesticide uptake in plants, we applied a plant PBK transfer model originally designed for fruit trees, supplemented by the Trapp model in regions lacking plant data. This dual-model approach addresses data gaps but introduces uncertainty, particularly for wild herbaceous species, and requires further refinement.

The results highlight regions with relatively higher contamination and risk estimates within the compiled dataset, but these findings are constrained by limited geographic coverage and should be interpreted as indicative patterns rather than definitive rankings. Elevated hazard and risk estimates in ecologically protected areas reveal critical gaps in pesticide monitoring, especially in wildlife tissues and remote ecosystems. Future assessments should prioritize systematic biomonitoring of wildlife across trophic gradients, integration of PBK modeling with isotopic tracing, increased reporting of pesticide residues from data-sparse regions, and linkage of ecological and toxicological databases to enable dynamic, predictive assessments. Empirical validation and expanded monitoring remain essential for global implementation.

## Resource availability

### Lead contact

Further information and requests for resources should be directed to and will be fulfilled by the lead contact, Prof. Zijian Li (lizijian3@mail.sysu.edu.cn).

### Materials availability

This study did not generate new unique reagents.

### Data and code availability

• All data in this study are reported in the supplemental information and will be publicly available upon publication.
• This paper does not report custom code.
• Any additional information required to reanalyze the data reported in this paper is available from the lead contact upon request.

## Acknowledgments

This study was financially supported by the 10.13039/501100001809National Natural Science Foundation of China (grant no. 32472598) and the Shenzhen Science and Technology Program (grant no. JCYJ20250604174437049).

## Author contributions

S.C.: conceptualization, methodology, data curation, writing – original draft, and writing – review and editing. Z.L.: conceptualization, methodology, writing – review and editing, and funding acquisition.

## Declaration of interests

The authors declare no competing interests.

## Declaration of generative AI and AI-assisted technologies in the writing process

During the preparation of this work, the authors used DeepSeek and ChatGPT in order to improve readability and language. After using this tool/service, the authors reviewed and edited the content as needed and take full responsibility for the content of the publication.

## STAR★Methods

### Key resources table

**Table:** 

REAGENT or RESOURCE	SOURCE	IDENTIFIER
**Software and algorithms**		

R version 4.2.2	R Core Team	https://www.r-project.org/
ArcGIS 10.8	Environmental Systems Research Institute	https://www.arcgis.com/index.html; RRID SCR_011081
Microsoft Excel 2021	Microsoft	https://excel.cloud.microsoft/
Origin 2024	OriginLab Corporation	https://www.originlab.com/; RRID: SCR_014212

**Other**		

Any additional information required to reanalyze the data reported in this paper	Available by request from lead contact	N/A

### Method details

#### General method

The overall framework used to investigate pesticide transfer across terrestrial food webs is illustrated in Scheme S1. First, we collected data on pesticide concentrations in wildlife livers, wild plants, and natural soils from various countries worldwide. To quantify the relative contamination levels across environmental matrices, we employed a unified scoring approach. Using these contamination scores, we traced pesticide propagation through the food chain from abiotic media to primary producers and then to consumers at successive trophic levels. To estimate wildlife exposure under data-sparse conditions, we implemented a tiered strategy (Tiers 1–3) that combines empirical contamination data, physiologically based pharmacokinetic (PBK) modeling, and soil-plant transfer models. Finally, we conducted non-carcinogenic and carcinogenic risk assessments using international toxicological benchmarks and mapped spatial patterns of ecological risk.

#### Data collection and inclusion criteria

To assemble a comprehensive dataset of pesticide concentrations in terrestrial wildlife, soils, and wild plants, we conducted a systematic literature search using the Web of Science database, covering publications from January 1, 2000, to December 31, 2024. Detailed search strategies are provided in the supplemental information.

Only studies reporting empirical measurements of pesticide concentrations were included; review articles, meta-analyses, modeling studies, and secondary analyses were excluded. To ensure ecological relevance, we excluded studies conducted in agricultural fields, managed farmlands, orchards, or areas subjected to pesticide application, retaining only samples from natural, non-degraded, and non-urbanized environments. Wild mammals were categorized into herbivores, carnivores, and omnivores. Only studies reporting liver concentrations were included, as the liver is the most consistently reported organ and a biologically informative site of accumulation; when multiple organs were reported, liver data were preferentially extracted.

After the application of all selection and quality-control criteria, a total of 100 studies from 30 countries were included in the final dataset, comprising 15 on herbivores, 13 on carnivores, 10 on omnivores, 47 on soils, and 15 on wild plants. Data on pesticide concentrations in animal organs and muscle tissues were extracted and standardized across studies. Concentrations in animal organs were uniformly expressed as nanograms per gram of lipid weight (ng⋅g^−1^ lipid weight), while soil and plant concentrations were reported as nanograms per gram of dry weight (ng⋅g^−1^) and nanograms per gram (ng⋅g^−1^), respectively. In cases where lipid normalization was not provided, conversions were performed using species-specific lipid data to ensure consistency across datasets. Each pesticide compound was subsequently identified and assigned a unique Chemical Abstracts Service (CAS) number based on the PubChem database, ensuring the accuracy and traceability of all chemical information.

#### Global contamination scores

Contamination scores (S) quantify deviations from global pesticide concentration means for soil, plants, and wildlife (Equation 1), with S>0 indicating above-average contamination (S is a comparative, non-toxicological index). For each matrix (wildlife liver, soil, or wild plant), the score for a given country is calculated as:

Equation 1 $$S=\frac{1}{M}\sum_{m=1}^{M}\left\{\frac{1}{N}\sum_{N=1}^{N}\left[\mathrm{Log}\left[C_{n,m}\times I\left(C_{n,m}\right)+\text{LOD}\times I\left(\text{LOD}\right)\right]-\bar{\log\;C_{n}}\right]\right\}\forall \;M\geq 1,N\geq 1$$

where S denotes the dimensionless contamination score for a specific environmental matrix (animal liver, soil, or plant) in each country. When S≤0, it signifies that pesticide levels are essentially similar to or below the global average. Conversely, when S>0, it signifies significantly higher pesticide levels in comparison to the global average. These thresholds are adjustable depending on future research focus and data scale. Importantly, S is a relative index and does not correspond to toxicological safety thresholds or risk limits. The symbol *Log*[C_n,m_ × I(C_n,m_) + LOD × I(LOD)] denotes the log (10) transformed value of the pesticide concentration (n) at sampling point (m) in the country. C_n,m_ represents the concentration of pesticide n at sampling point m, and I(C_n,m_) and I(LOD) are indicator functions defined as:

Equation 2 $$I\left(C_{n,m}\right)=\left\{\begin{array}{cc} 1\text{;} & \text{if}\;\text{detectable}\\ 0\text{;} & \text{if}\;\text{undetectable} \end{array}\right\};\;I\left(\text{LOD}\right)=\left\{\begin{array}{cc} 0\text{;} & \text{if}\;\text{detectable}\\ 1\text{;} & \text{if}\;\text{undetectable} \end{array}\right\}$$

LOD represents the analytical limit of detection; where unavailable, the limit of quantification (LOQ) is used. The term $\bar{\log\;C_{n}}$ denotes the global central tendency of pesticide n, computed as:

Equation 3 $$\bar{\log\;C_{n}}=\frac{1}{Q}\sum_{q=1}^{Q}\log\;C_{n}$$

where Q is the total number of global sampling points across all countries. *C*_*n*_ denotes the concentration of pesticide n in a specific species within a given country/region. To achieve comparability across environmental compartments, all concentrations were standardized before analysis.

For each sampling site, we take the log-transformed concentration (substituting LOD for non-detects) and subtract the global mean of that pesticide. This deviation is then averaged first over all pesticides detected at the site, then over all sites within the country. A positive S indicates that the country’s contamination level tends to be above the global average; S≤0 suggests levels near or below the average. We calculated separate scores for soil (*S*_*Soil*_), wild plant (*S*_*wildplant*_), and each animal guild (*S*_*animal*_). These scores allow us to trace how contamination propagates through the food chain: from abiotic media (soil) to primary producers (plants) and then to consumers at successive trophic levels.

#### Tiered strategy for estimating wildlife exposure under data-sparse conditions

Empirical concentration data are not uniformly available across all countries or trophic levels. To maximize the use of existing information while maintaining methodological consistency, we implemented a hierarchical (tiered) approach. The guiding principle is to rely on the most direct data available, and to introduce model-based extrapolation only when necessary. Uncertainty increases from Tier 1 to Tier 3, and this is propagated through subsequent risk calculations.

Tier 1 (most direct): Measured concentrations in wildlife tissues (liver) are available → we use PBK-based reverse dosimetry to estimate the average daily dose (ADD) directly.

Tier 2 (intermediate): Wildlife tissue data are absent, but measured plant concentrations exist → we estimate herbivore exposure via plant ingestion, and then derive carnivore and omnivore exposure through food-chain transfer.

Tier 3 (least direct): Only soil concentration data are available → we first simulate plant concentrations using a soil-plant transfer model, then follow the Tier-2 procedure to propagate exposure upward.

The following sections describe each tier in detail. Prior to all calculations, concentrations from raw data were consistently converted to mg·kg^-1^ wet weight for animal tissues and mg·kg^-1^ for plant and soil matrices. Specifically, concentrations originally reported as nanograms per gram of lipid weight (ng⋅g^−1^ lipid weight) for animal tissues were converted to mg kg-1 wet weight using species-specific lipid content (*f*_lipid_) and a unit conversion factor. Concentrations for soil and wild plants (ng⋅g^−1^) were converted to mg·kg^-1^ by multiplying by 10^-3^. This ensures all subsequent calculations are performed with consistent units.

#### Tier 1: Using PBK models to back-calculate pesticide exposure in animals

To estimate dietary exposure to pesticides in animals, we used mass-adjusted biotransformation factors (BTF), defined as the extent to which orally ingested pesticides are transformed within tissues. By applying BTF values to measured tissue concentrations, we reconstructed the average daily dose (ADD, mg⋅kg^−1^day^-1^) for each species, enabling precise cross-species comparisons across trophic levels.^18^ The BTF (mg⋅kg^−1^ per mg⋅kg^−1^) represents the vector of biotransformation factors for that chemical in tissues and organs via oral exposure pathways. The general mathematical expression for BTF is:

Equation 4 $${BTF}_{species,tissue\;}=\frac{C_{species,tissue\;}}{{Intake}_{species\;}}$$

In this study, *C*_*species*,*tissue*_ is used to denote the tissue concentration of species under steady-state conditions (mg⋅kg^−1^), *Intake*_*species*_ denotes the amount of pesticides accumulated by a species through food intake (mg⋅day^−1^).*Intake*_*speciesn*_ can then be calculated by rearranging the BTF Equation 4:

Equation 5 $${Intake}_{species\;}=\frac{C_{species,tissue\;}}{{BTF}_{species,tissue\;}}$$

To assess animal exposure to various pesticides through dietary intake, average daily exposure levels (*ADD*_*species*_) were estimated. *ADD*_*species*_ denotes the weight-standardized external exposure dose under steady-state conditions (mg⋅kg^−1^⋅day^−1^), calculated as:

Equation 6 $${ADD}_{species\;}=\frac{{Intake}_{species\;}}{{BW}_{species\;}}$$

Where *BW*_*species*_ denotes represents the body weight of species (kg). Combining Equations 5 and 6 yields the following result:

Equation 7 $${ADD}_{species\;}=\frac{C_{species,tissue\;}}{{BTF}_{species,tissue\;}\times {BW}_{species\;}}$$

ADD for herbivores, carnivores, and omnivores was calculated via Equations 8, 9, and 10, using species-specific BTFs and physiological parameters (Table S2–S23). The average daily pesticide exposure for carnivores, omnivores, and herbivores is as follows:

Equation 8 $${ADD}_{\text{herbivores},1}=\frac{C_{\text{herbivores},\text{tissue}}}{{BTF}_{\text{herbivores},\text{tissue}}\times {BW}_{\text{herbivores}}}$$

Equation 9 $${ADD}_{\text{carnivores},1}=\frac{C_{\text{carnivores},\text{tissue}}}{{BTF}_{\text{carnivores},\text{tissue}}{\times BW}_{\text{carnivores}}}$$

Equation 10 $${ADD}_{\text{omnivores},1}=\frac{C_{\text{omnivores},\text{tissue}}}{{BTF}_{\text{omnivores},\text{tissue}}{\times BW}_{\text{omnivores}}}$$

#### Tier 2: Determining the external exposure dose of herbivores, carnivores and omnivores using plant concentrations

In countries lacking wildlife tissue data but with measured plant concentrations, we estimated herbivore exposure directly from plant ingestion and propagated concentrations to higher trophic levels using dietary transfer equations, using measured plant concentrations as proxy data.

For herbivores, the daily intake is calculated as the product of the measured plant concentration and the herbivore’s *Intake*_herbivores_:

Equation 11 $${Intake}_{\text{herbivores}}=C_{wild\;plant}\cdot {IR}_{\text{herbivores}}$$

Consequently, the Average Daily Dose for herbivores (*ADD*_herbivores,2_) can be calculated by integrating Equations 6 and 11.

Equation 12 $${ADD}_{\text{herbivores},2}=\frac{C_{wild\;plant}\cdot {IR}_{\text{herbivores}}}{{BW}_{\text{herbivores}}}$$

*C*_*wildplant*_ (mg⋅kg^−1^) denotes the concentration of wild plants in the collected sample, incorporating leaves, stems, grasses, roots and seeds. However, pollen and nectar are excluded from calculation. The values in the dataset are presented in the excel file (see details therein). *IR*_herbivores_ denotes the daily intake rate of herbivores, measured in kilograms per day (kg⋅day^−1^). The calculation method for *BW*_herbivores_ denotes body weight of herbivores, measured in kilograms (kg).

The pesticide concentration in herbivore tissues (*C*_herbivores, tissue_, mg⋅kg^−1^) is calculated based on the BTF of the herbivore tissue, the daily plant intake of the herbivore, and the plant concentration, as detailed in Equation 13.

Equation 13 $$C_{\text{herbivores},\text{tissue}\;}={BTF}_{\text{herbivores},\text{tissue}\;}\times {IR}_{\text{herbivores}}\times C_{wild\;plant}$$

where *C*_*wildplant*_ is as defined above. Carnivores and omnivores consume specific tissues (typically muscle and fat) from herbivores. When extrapolating from plant food sources, the concentration value for that specific tissue can be obtained using the BTF for a single tissue from herbivores. To derive the average intake concentration for carnivores, a weighted average calculation across all tissues of herbivores is required.

Equation 14 $${\bar{C}}_{\text{herbivores}}=\frac{\sum_{i=1}^{n}\left(C_{\text{herbivores},\text{tissue}}\times w_{tissuet}\right)}{\sum_{i=1}^{n}w_{tissue}}$$

${\bar{C}}_{\text{herbivores}}$ (mg⋅kg^−1^) represents the weighted average of concentrations in herbivores.*w*_*tissue*_ represents the weighting factor for a certain tissue within the body, as previously addressed in extant studies.^54^ *C*_herbivores,tissue_ denote the concentration of tissue (muscle and fat). n stands for the total number of data points. Through the consumption of herbivores, the dose of external exposure to which carnivores are subjected can be determined (*ADD*_carnivores,2_).

Equation 15 $${ADD}_{\text{carnivores},2}=\frac{{\bar{C}}_{\text{herbivores}}{\times IR}_{\text{carnivores}}}{{BW}_{\text{carnivores}}}$$

Utilizing the concept of the weighted average concentration of herbivores, it is possible to calculate the concentration in carnivores through the food chain.

Equation 16 $$C_{\text{carnivores},\text{tissue}\;}={BTF}_{\text{carnivores},\text{tissue}\;}{\times IR}_{\text{carnivores}}\times {\bar{C}}_{\text{herbivores}}$$

*BTF*_carnivores, tissue_ can be obtained through previous research. Detailed specifications can be found in the supplemental information. *IR*_carnivores_ represents the daily intake of carnivores. As a food source for omnivores, the weighted average concentration of carnivores (${\bar{C}}_{\text{carnivores}}$) must be calculated when determining the external exposure dose for omnivores. The calculation method is similar to Equation 14, as detailed in Equation 17.

Equation 17 $${\bar{C}}_{\text{carnivores}}=\frac{\sum_{i=1}^{n}\left(C_{\text{carnivores},\text{tissue}}\times w_{tissue}\right)}{\sum_{i=1}^{n}w_{tissue}}$$

${\bar{C}}_{\text{carnivores}}$ (mg⋅kg^−1^) represents the weighted average of concentrations in carnivores. *w*_*tissue*_ is the tissue weighting factor as defined in Equation 14. *C*_carnivores, tissue_ denote the concentration of tissue (muscle and fat).

In this study, adjustments were made based on species feeding characteristics in order to infer the level of external exposure experienced by omnivores (*ADD*_omnivores,1_).

Equation 18 $${ADD}_{\text{omnivores},2}=\frac{\left(f_{p}.C_{plant}+f_{h}{\cdot \bar{C}}_{\text{herbivores}}{+f}_{c}{\cdot \bar{C}}_{\text{carnivores}}\right)\cdot {IR}_{\text{omnivores}}}{{BW}_{omnivores}}$$

*f*_*p*_ denotes the proportion of plant-based foods in total intake, ranging from 0 to 1. *f*_*h*_ represents the weight of herbivores in total intake, ranging from 0 to 1. *f*_*c*_ is used to represent the weight of carnivores in total intake, ranging from 0 to 1. The sum of *f*_*p*_, *f*_*h*_, and *f*_*c*_ does not exceed 1 and is allocated according to the specific distribution of species in the supplemental information. *IR*_omnivores_ represents the daily food intake of omnivores (kg⋅day^−1^). *BW*_omnivores_ represents the body weight (kg) of omnivorous animals.

#### Tier 3: Deriving external exposure doses for herbivores, carnivores and omnivores using soil concentrations

For countries with only soil data, the soil-plant transfer model is used to simulate pesticide concentrations in plants through modeling^55^:

Equation 19 $$C_{Plant\;model}=C_{soil}\times {BAF}_{plant}^{H}\left(T_{Air},{RH}_{Air}\right)$$

Equation 20 $${BAF}_{plant}^{H}\left(T_{Air},{RH}_{Air}\right)=\frac{Q^{H}\left(T_{Air},{RH}_{Air}\right)\times \frac{1}{k_{d}}}{A_{L}^{H}\cdot g\cdot \frac{1000}{K_{LA}}+\lambda^{H}\cdot M_{L}^{H}}$$

*C*_*soil*_ represents the pesticide concentration in soil (mg⋅kg^−1^). The bioaccumulation factor for herbaceous plant leaves, denoted as ${BAF}_{plant}^{H}\;\left(T_{Air},{RH}_{Air}\right)$, is dimensionless and represents the ratio of plant concentration to soil concentration. *Q*^*H*^(*T*_*Air*_, *RH*_*Air*_) represents the transpiration rate per leaf, which is a function of air temperature and relative humidity. *k*_*d*_ denotes the soil-water distribution coefficient. $A_{L}^{H}$ represents the leaf surface area. g denotes vapor conduction. *K*_*LA*_ represents the leaf-air distribution coefficient. *λ*^*H*^ indicates the total loss rate of pesticides within leaves, encompassing photodegradation, plant metabolism, and dilution due to plant growth. $M_{L}^{H}$ denotes the mass of a single leaf. The soil-to-plant transfer model follows the mechanistic structure proposed by Trapp’s research, assuming steady-state diffusion and transpiration-driven uptake.^56^ For specific calculation details, please refer to the supplemental information (Table S1 and Method S1 and S2).

Concentrations in plants derived from soil concentrations, which are then used to estimate the ADD of herbivores (*ADD*_herbivores,3_) in a given region.

Equation 21 $${ADD}_{\text{herbivores},3}=\frac{C_{\text{Plant}\;m\text{odel}}\cdot {IR}_{\text{herbivores}}}{{BW}_{\text{herbivores}}}$$

Substituting *C*_*plantmodel*_ for *C*_*wildplant*_ in Equation 13 yields the simulated herbivore tissue concentration, denoted as *C*_herbivores, tissue,PM_. The calculation of the external exposure of carnivores through soil simulation depends on the weighted average concentration ${\bar{C}}_{\text{herbivores},\text{PM}}$. This concentration is obtained by substituting *C*_herbivores,tissue,PM_ into Equation 14, thereby yield *ADD*_carnivores,3_.

Equation 22 $${ADD}_{\text{carnivores},3}=\frac{{\bar{C}}_{\text{herbivores},\text{PM}}{\times IR}_{\text{carnivores}}}{{BW}_{\text{carnivores}}}$$

Substituting ${\bar{C}}_{\text{herbivores},\text{PM}}$ into Equation 16 gives *C*_carnivores,tissue,PM_, which is then used in Equation 17 to obtain *C*_carnivores, tissue,PM_.The result *C*_carnivores, tissue,PM_ should be substituted as the value for *C*_carnivores,PM_. from Equation 17 in order to obtain ${\bar{C}}_{\text{carnivores},\text{PM}}$. The external exposure levels of omnivorous animals (*ADD*_omnivores,3_) obtained from soil are demonstrated in Equation 23.

Equation 23 $${ADD}_{\text{omnivores},3}=\frac{\left(f_{p}.C_{plant\;model}+f_{h}{\cdot \bar{C}}_{\text{herbivores},\text{PM}}{+f}_{c}{\cdot \bar{C}}_{\text{carnivores},\text{PM}}\right)\cdot {IR}_{\text{omnivores}}}{{BW}_{omnivores}}$$

#### Exposure and ecological risk assessments

Non-carcinogenic and carcinogenic risks to wildlife were assessed following the USEPA health risk assessment framework, using toxicity parameters from the Integrated Risk Information System (IRIS) and relevant literature. Toxicity data for chemical substances, including oral reference doses (RfD, mg·kg^-1^·day^-1^) and oral slope factors (OSF, per mg·kg^-1^·day^-1^), are primarily sourced from the USEPA’s IRIS database and other authoritative toxicological materials. For each substance, the most sensitive toxicity endpoint data are prioritized for selection.

Specifically: Non-carcinogenic risk assessment: The RfD is used, representing the estimated acceptable daily dose for lifetime exposure below which no appreciable non-carcinogenic adverse effects are expected. HQ is defined as the non-carcinogenic risk to species health from long-term exposure to a given chemical substance.

Equation 24 $$HQ=\frac{ADD}{RfD}$$

ADD values were estimated as described in Tiers 1–3. It is important to note that we prioritized concentration data from animals, the most accurate source, and used measured or modeled plant concentrations only as a supplement when animal concentrations were unavailable for a given country. Typically, HQ≤1 indicates that non-carcinogenic risks are negligible or insignificant; HQ>1 suggests potential non-carcinogenic risks may exist, with higher values indicating greater risk levels.

Carcinogenic Risk Assessment: The OSF is employed, representing the upper bound estimate of lifetime carcinogenic risk resulting from exposure to a unit dose of carcinogen over a lifetime. ECR indicates the carcinogenic risk to species health from long-term exposure to a chemical substance.

Equation 25 ECR=ADD×OSF

In general, an ECR of≤1×10^-6^ is considered to pose a negligible risk, while an ECR of >1×10^-4^ is deemed to carry an unacceptable risk. Risk levels falling between these two thresholds require careful judgement based on the specific research context.

The screening of target chemicals identified 34 pesticides with established toxicological parameters (Table S24). The ADD values estimated for the three trophic groups were compared against the corresponding toxicity thresholds to characterize potential health risks across different feeding guilds.

The spatial distribution of chemical pollution and its associated ecological risks was visualized using ArcGIS 10.8. National-level pollution scores (*S*_*animal*_, *S*_*wildplant*_,*S*_*soil*_) were linked to global administrative boundary shapefiles (Table S25). Three thematic maps were generated: (i) pollution intensity map, (ii) HQ map, and (iii) ECR map (Figures 4, 5, and 6).

#### Data quality and uncertainty analysis

To assess uncertainty and variability in risk estimation, this study employed Monte Carlo simulation methods to conduct probabilistic analysis of HQ for representative persistent organic pollutants (POPs) and modern pesticides. All input parameters, including measured tissue concentrations, physicochemical properties (e.g., Log Kow, Log Kaw, half-life), and physiological parameters (e.g., feeding rate, body weight), were assumed to follow a log-normal distribution to reflect the typical skewness characteristic of environmental contaminant concentrations. The Monte Carlo simulation ran for 10,000 iterations to generate probability density distributions of HQ values for each chemical across different trophic levels. The simulation results were used to assess the robustness of risk estimates and evaluate model performance by comparing distributions with available data. Specifically, the parameter distributions used in the simulations were based on statistical characteristics from literature reports or measured data. For parameters where explicit variability was not available, a coefficient of variation of 30% was assumed.

To identify parameters that most influence HQ estimates, a sensitivity analysis using Pearson correlation was conducted. Parameters were considered to have a significant effect on model outputs if |ρ|≥0.4.

To quantify the contribution of each input parameter to the variance in ECR estimates, we performed Sobol sensitivity analysis using a case study of p,p′-DDE in foxes, considering both the soil-derived pathway (Tier 3) and the direct tissue-based pathway (Tier 1). The Sobol method decomposes the output variance into first-order effects (direct contribution of a single parameter) and total-order effects (direct plus interaction effects). The first-order index (S1) measures the fraction of variance explained by a parameter alone; the total-effect index (ST) measures the parameter’s overall contribution, including interactions with others. A large difference between ST and S1 (ST - S1) indicates strong interactions. Parameters with high ST values are the primary drivers of model uncertainty and should be prioritized for more precise measurement in future studies. For the tissue-based pathway, 2,000 iterations were performed; for the soil-based pathway, 10,000 iterations were used to ensure convergence. Confidence intervals (95%) for sensitivity indices were obtained by bootstrapping.

### Quantification and statistical analysis

The exact values of n (number of sampling points, number of studies, or number of species) are reported in the results section and figure legends. n represents the number of independent measurements for each matrix and chemical. Measures of central tendency and dispersion (mean, median, standard deviation) are reported where applicable. No statistical hypothesis tests were performed, as all figures are intended for descriptive visualization only.

## Supplemental information

Document S1. Figures S1 and S2, Scheme S1, Tables S1–S26, and Methods S1 and S2

Data S1. Dataset of ecological carcinogenic risk (ECR) and hazard quotient (HQ) values used for ecological risk assessment and Monte Carlo simulation, related to Figures 2, 3, and 5 (A) Biological transfer factors (BTFs) for various animal tissues. (B) ECR and HQ derived from PBK models. (C) ECR and HQ calculated from plant concentrations. (D) ECR and HQ calculated using soil concentrations and plant models. (E) Reference dose (RfD) and oral slope factor (OSF) for the chemicals covered in the article. (F) Physiological parameters for various animal species.

Data S2. Dataset of contamination scores for soil, wild plants, and animals across countries, related to Figure 1 (A) Summary of liver concentrations in carnivores. (B) Summary of liver concentrations in herbivores. (C) Summary of liver concentrations in omnivores. (D) Summary of concentrations in wild plants. (E) Summary of soil concentrations.
