# Supplementary material for: Tracing pesticides through terrestrial food webs with wildlife at risk
Source: iScience. 2026 Apr 22;29(6):115870. doi: 10.1016/j.isci.2026.115870 (PMC13156712; doi:10.1016/j.isci.2026.115870)
Supplement: Document S1. Figures S1 and S2, Scheme S1, Tables S1–S26, and Methods S1 and S2 [file mmc1.pdf]

iScience, Volume *29*

Supplemental information

Tracing pesticides through terrestrial food webs with wildlife at risk

Shaorong Chen and Zijian Li

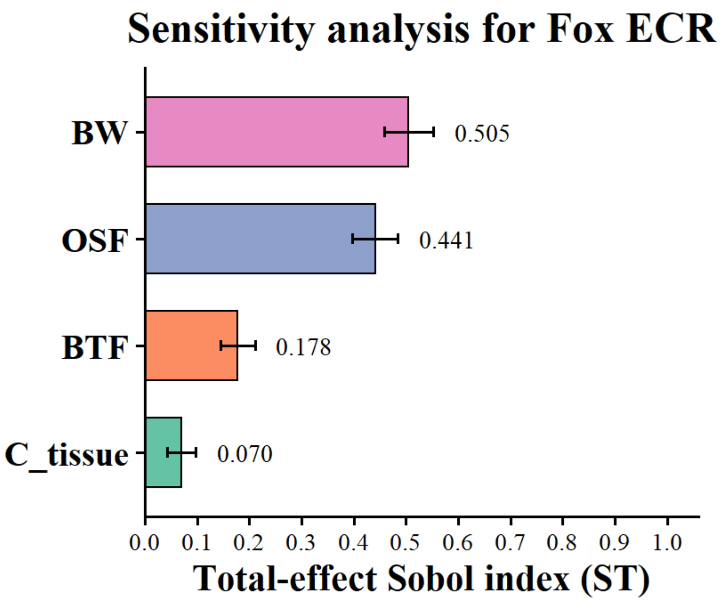

Figure S1. Global Sensitivity Ranking of p,p'-DDE for Ecological Risk Assessment in Foxes (Based on Liver Data), Related to the Results (“Uncertainty and interpretation of estimated ecological health risks”)

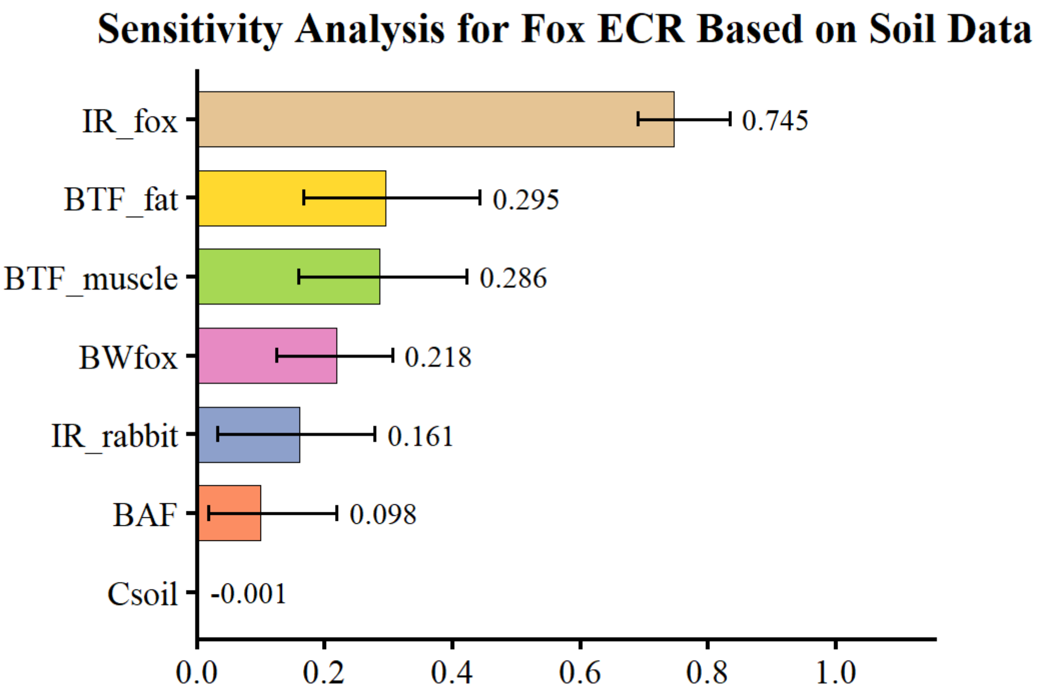

Figure S2. Global Sensitivity Ranking of p,p'-DDE for Ecological Risk Assessment in Foxes (Based on Soil Data), Related to the Results ("Uncertainty and interpretation of estimated ecological health risks")

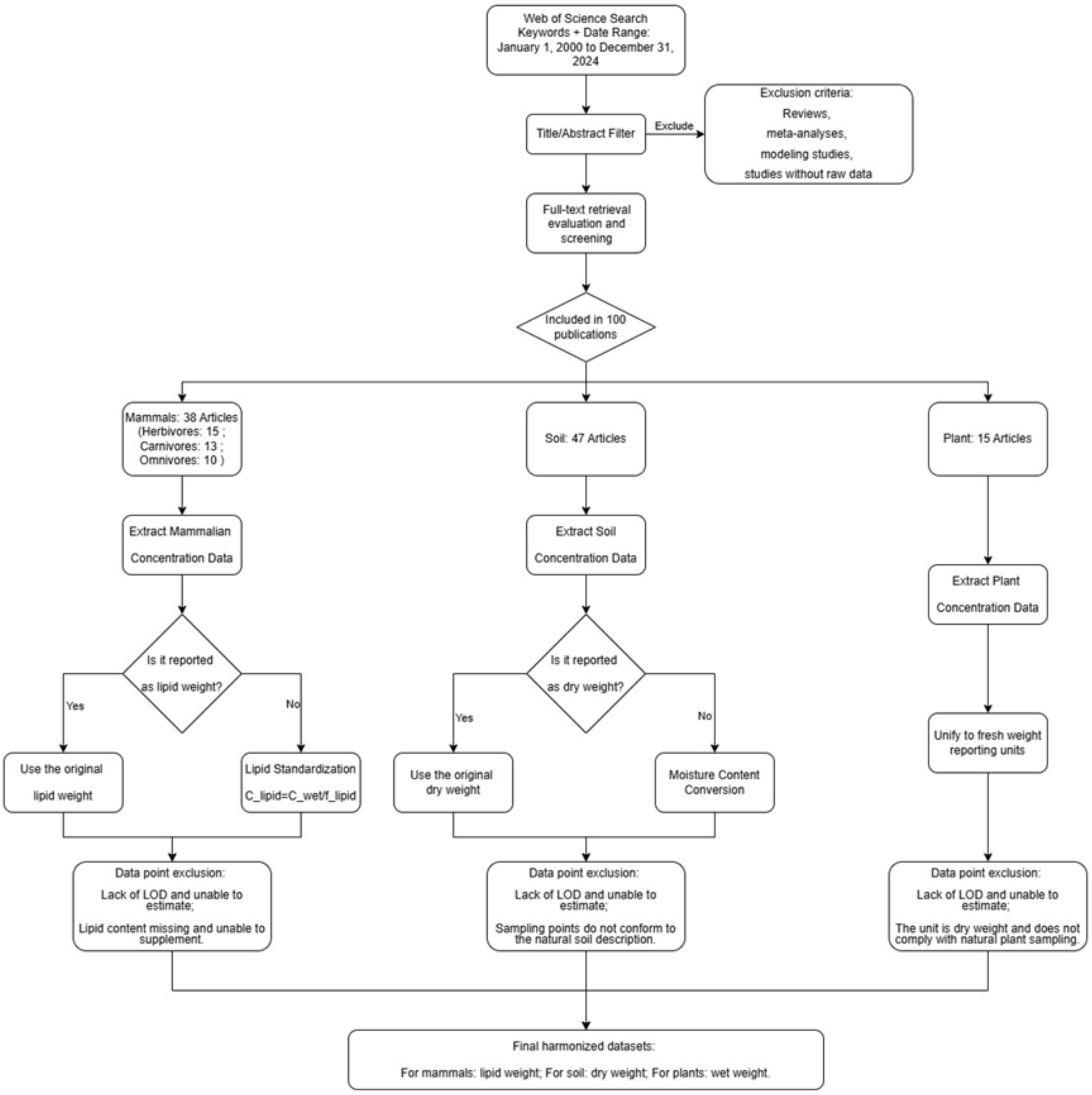

Scheme S1. Flow chart of the methodology in this study, Related to the STAR Methods ("General method")

Table S1. Summarizes parameters used in the transpiration rate model and herbaceous plants (leaves) ,Related to the STAR Methods (“Tiered strategy for estimating wildlife exposure under data-sparse conditions”).

**Parameter**	**Symbol**	**Unit**	**Value**
Leaf mass per area	LMA_L^H	$kg \cdot m^2$	0.05
Conductance	g	$m \cdot d^{-1}$	86.4
Water content of leaf	W_L	$L \cdot kg^{-1}$	0.8
Lipid content of leaf	L_L	$kg \cdot kg^{-1}$	0.02
Corrected coefficient	cc	unitless	0.95
Density of octanol	$\rho_{Octanol}$	$kg \cdot L^{-1}$	0.83
Organic carbon content in soil	f_{oc}	$g \cdot g^{-1}$	1.23×10^{-2}
Total pesticide loss rate in the leaves	λ_L^H	day^{-1}	0.095
Density of air	ρ_{Air}	$kg \cdot m^3$	1.225
Density of water	ρ_{Water}	$kg \cdot L^{-1}$	1
Leaf area index	LAI	m^2m^{-2}	0.22
Specific heat of air	c_p	$kJ \cdot kg^{-1}K$	1.005
Latent heat of water vaporization	ΔH_{Vap}	$kJ \cdot kg^{-1}$	2260
Psychrometric constant	γ	$Pa \cdot K^{-1}$	66.1
Conductance	g	$m \cdot d^{-1}$	86.4
Ideal gas constant	R	$kJ \cdot K^{-1}mol^{-1}$	0.008314
Stomatal resistance	R_s	$s \cdot m^{-1}$	200
Aerodynamic drag	R_{Air}	$s \cdot m^{-1}$	83.53

Table S2. Summary of physiological parameters of Moose, Related to the STAR Methods (" Tiered strategy for estimating wildlife exposure under data-sparse conditions").

Physiological variables	Symbol	Unit	Value	Reference
Food intake rate	$IR_{n \to n+1,oral}$	Kg day^{-1}	20.00	Estimated
Exhalation rate	$ER_{Exhalation}$	kg day^{-1}	483.84	(Hugh-Jones et al., 1978; Siegal-Willott et al., 2009)
Urine excretion rate	ER_{Urine}	kg day^{-1}	23.00	Estimated
Bile excretion rate	ER_{Bile}	kg day^{-1}	7.58	Estimated.(DelGiudice et al., 1995)
Body mass	---	kg	700.00	(Schladweiler & Stevens, 1973)
Blood mass	M_{blood}	kg	33.60	Estimated
Liver mass	M_{liver}	kg	4.14	(Jensen et al., 2013)
Kidney mass	M_{kidney}	kg	1.89	(Jensen et al., 2013)
Lung mass	M_{lung}	kg	1.94	Estimated
Fat mass	M_{fat}	kg	98.00	Estimated
Muscle mass	M_{muscle}	kg	336.00	(Jensen et al., 2013)
Blood flow rate of liver	$Q_{Blood \Leftrightarrow Liver}$	kg day^{-1}	13353.98	Estimated
Blood flow rate of kidney	$Q_{Blood \Leftrightarrow Kidney}$	kg day^{-1}	967.68	Estimated
Blood flow rate of lung	$Q_{Blood \Leftrightarrow Lung}$	kg day^{-1}	4612.61	Estimated
Blood flow rate of fat	$Q_{Blood \Leftrightarrow Fat}$	kg day^{-1}	741.89	Estimated
Blood flow rate of muscle	$Q_{Blood \Leftrightarrow Muscle}$	kg day^{-1}	10708.99	Estimated

Table S3.Summary of composition of Moose tissues, Related to the STAR Methods ("Tiered strategy for estimating wildlife exposure under data-sparse conditions").

Media/ Products	Contents (g g^{-1})				References
	Lipid (fat)	Non-lipid organics	Water	Others	
Blood	0.0075	0.08	0.91	0.0025	Estimated
Urine	0	0	0.95	0.05	(Gulhane et al., 2017a)
Bile	0.0056	0.0004	0.894	0.1	(*My Food Data - Free Nutrition Tools to Understand What You Eat*, n.d.-a)
Liver	0.1029	0.1997	0.6788	0.0186	(Reimers et al., 1982)
Lung	0.026	0.167	0.797	0.011	(*My Food Data - Free Nutrition Tools to Understand What You Eat*, n.d.-a)
Kidney	0.03	0.165	0.792	0.013	(*My Food Data - Free Nutrition Tools to Understand What You Eat*, n.d.-a)
Fat	0.85	0	0.15	0	(Reimers et al., 1982)
Muscle	0.0492	0.1958	0.7462	0.0088	(Reimers et al., 1982)

Table S4. Summary of physiological parameters of Caribous, Related to the STAR Methods (“ Tiered strategy for estimating wildlife exposure under data-sparse conditions”).

Physiological variables	Symbol	Unit	Value	Reference
Food intake rate	$IR_{n\rightarrow n+1,oral}$	kg day^{-1}	5.00	Estimated
Exhalation rate	$ER_{Exhalation}$	kg day^{-1}	50.35	Estimated
Urine excretion rate	ER_{Urine}	kg day^{-1}	2.83	Estimated
Bile excretion rate	ER_{Bile}	kg day^{-1}	1.50	Estimated. (DelGiudice et al., 1995)
Body mass	---	kg	155.00	(Gerhart et al., 1996)
Blood mass	M_{blood}	kg	12.87	Estimated
Liver mass	M_{liver}	kg	3.10	(Adamczewski et al., 1995)
Kidney mass	M_{kidney}	kg	0.42	(Adamczewski et al., 1995)
Lung mass	M_{lung}	kg	2.79	Estimated
Fat mass	M_{fat}	kg	11.83	Estimated
Muscle mass	M_{muscle}	kg	74.40	(Adamczewski et al., 1995)
Blood flow rate of liver	$Q_{Blood\Leftrightarrow Liver}$	kg day^{-1}	6801.19	Estimated
Blood flow rate of kidney	$Q_{Blood\Leftrightarrow Kidney}$	kg day^{-1}	492.84	Estimated
Blood flow rate of lung	$Q_{Blood\Leftrightarrow Lung}$	kg day^{-1}	2349.20	Estimated
Blood flow rate of fat	$Q_{Blood\Leftrightarrow Fat}$	kg day^{-1}	377.84	Estimated
Blood flow rate of muscle	$Q_{Blood\Leftrightarrow Muscle}$	kg day^{-1}	5454.10	Estimated

Table S5. Summary of composition of Caribous tissues, related to the STAR Methods ("Tiered strategy for estimating wildlife exposure under data-sparse conditions").

Media/Products	Contents ($g\ g^{-1}$)				References
	Lipid (fat)	Non-lipid organics	Water	Others	
Blood	0.0075	0.08	0.91	0.0025	Estimated
Urine	0	0	0.95	0.05	(Gulhane et al., 2017a)
Bile	0.0056	0.0004	0.894	0.1	(My Food Data - Free Nutrition Tools to Understand What You Eat, n.d.-a)
Liver	0.1029	0.1997	0.6788	0.0186	(Reimers et al., 1982)
Lung	0.026	0.167	0.797	0.011	(My Food Data - Free Nutrition Tools to Understand What You Eat, n.d.-a)
Kidney	0.03	0.165	0.792	0.013	(My Food Data - Free Nutrition Tools to Understand What You Eat, n.d.-a)
Fat	0.85	0	0.15	0	(Reimers et al., 1982)
Muscle	0.0492	0.1958	0.7462	0.0088	(Reimers et al., 1982)

Table S6. Summary of physiological parameters of Chiroptera, Related to the STAR Methods (“ Tiered strategy for estimating wildlife exposure under data-sparse conditions”).

Physiological variables	Symbol	Unit	Value	Reference
Food intake rate	$IR_{n\rightarrow n+1,oral}$	kg day^{-1}	0.0800	Estimated
Exhalation rate	$ER_{Exhalation}$	kg day^{-1}	1.0300	Estimated
Urine excretion rate	ER_{Urine}	kg day^{-1}	0.0100	Estimated
Bile excretion rate	ER_{Bile}	kg day^{-1}	0.0140	Estimated
Body mass	---	kg	0.1000	(Jürgens et al., 1981)
Blood mass	M_{blood}	kg	0.0100	Estimated
Liver mass	M_{liver}	kg	0.0064	(Jürgens et al., 1981)
Kidney mass	M_{kidney}	kg	0.0016	(Jürgens et al., 1981)
Lung mass	M_{lung}	kg	0.0011	Estimated
Fat mass	M_{fat}	kg	0.0100	Estimated
Muscle mass	M_{muscle}	kg	0.0400	(Jürgens et al., 1981)
Blood flow rate of liver	$Q_{Blood\Leftrightarrow Liver}$	kg day^{-1}	415.7500	Estimated
Blood flow rate of kidney	$Q_{Blood\Leftrightarrow Kidney}$	kg day^{-1}	582.0500	Estimated
Blood flow rate of lung	$Q_{Blood\Leftrightarrow Lung}$	kg day^{-1}	831.5000	Estimated
Blood flow rate of fat	$Q_{Blood\Leftrightarrow Fat}$	kg day^{-1}	166.3000	Estimated
Blood flow rate of muscle	$Q_{Blood\Leftrightarrow Muscle}$	kg day^{-1}	1496.7000	Estimated

Table S7.Summary of composition of Chiroptera tissues, Related to the STAR Methods ("Tiered strategy for estimating wildlife exposure under data-sparse conditions").

Media/Products	Contents (g g-1)				Reference
	Lipid (fat)	Non-lipid organics	Water	Others	
Blood	0.0010	0.0360	0.96	0.0030	Estimated
Urine	0.0000	0.0000	0.95	0.0500	Estimated
Bile	0.0056	0.0004	0.894	0.1000	Estimated
Liver	0.0610	0.2050	0.734	0.0000	Estimated
Kidney	0.0770	0.1670	0.756	0.0000	Estimated
Muscle	0.0260	0.2260	0.748	0.0000	Estimated
Fat	0.8550	0.0450	0.1	0.0000	Estimated
Lung	0.0530	0.1650	0.782	0.0000	Estimated

Table S8. Summary of physiological parameters of Cattle, Related to the STAR Methods ("Tiered strategy for estimating wildlife exposure under data-sparse conditions").

Symbol	Variables	Value	Unit	Note
$IR_{grass\rightarrow cattle,oral}$	Daily grass intake mass by Cattle	20	kg day^{-1}	Estimated (dry mass) based on 3% of the body mass (600 kg)
$ER_{Bile,Cattle}$	Biliary excretion rate	6.5	kg day^{-1}	(Symonds et al., 1982)and(Sutton, 1985)estimated the combined bile and pancreatic secretion rate of 13 L day^{-1}.
$ER_{Exhalation,Cattle}$	Expiratory rate	260	kg day^{-1}	Estimated from the tidal volume and the breath per minute of cattle with the air density of 0.0012 kg L^{-1}.(Donald G. Stevens, 1981)
$ER_{Urine,Cattle}$	Urinary excretion rate	20	kg day^{-1}	Estimated from the urine volume per body weight per day.(Ashara & Shah, 2016)
$ER_{Milk,Cattle}$	Milk excretion rate	32.6	kg day^{-1}	Estimated(Løvendahl & Sehested, 2016)
---	Cattle body weight	600	kg	Generic
$M_{Blood,Cattle}$	Cattle blood quality	22.8	kg	Estimated (3.8% of the body mass)(Lautz et al., 2020)
$M_{Liver,Cattle}$	Cattle liver quality	7.8	kg	Estimated (1.3% of the body mass)(Lautz et al., 2020)
$M_{Lung,Cattle}$	Cattle lung quality	4.8	kg	Estimated (0.8% of the body mass)(Lautz et al., 2020)
$M_{Kidney,Cattle}$	Cattle kidney quality	1.2	kg	Estimated (0.2% of the body mass) (Lautz et al., 2020)
$M_{Fat,Cattle}$	Cattle fat quality	110.4	kg	Estimated (adipose tissue) (Lautz et al., 2020)
$M_{Muscle,Cattle}$	Cattle muscle quality	240	kg	Estimated (40% of the body mass; averaged from dairy and beef cattle) (Lautz et al., 2020)
$M_{Mammary\ gland,Cattle}$	Cattle mammary gland quality	13.2	kg	Estimated (2.2% of the body mass) (Lautz et al., 2020)
$Q_{B\Leftrightarrow Liver,Cattle}$	Blood flow rate of compartment liver	56739	kg day^{-1}	Estimated from dairy cattle (sum of hepatic artery and portal vein flows)(Lautz et al., 2020)

$Q_{B\Leftrightarrow Lung,Cattle}$	Blood flow rate of compartment lung	2579	kg day^{-1}	Estimated from sheep (Lautz et al., 2020)
$Q_{B\Leftrightarrow Kidney,Cattle}$	Blood flow rate of compartment kidney	1375	kg day^{-1}	Estimated from dairy cattle(Lautz et al., 2020)
$Q_{B\Leftrightarrow Fat,Cattle}$	Blood flow rate of compartment fat	5846	kg day^{-1}	Estimated from dairy cattle(Lautz et al., 2020)
$Q_{B\Leftrightarrow Muscle,Cattle}$	Blood flow rate of compartment muscle	1633	kg day^{-1}	Estimated from dairy cattle (Lautz et al., 2020)
$Q_{B\Leftrightarrow Mammary,Cattle}$	Blood flow rate of compartment mammary gland	14185	kg day^{-1}	Estimated from dairy cattle(Lautz et al., 2020)

Table S9. Summary of nutritional composition of cattle tissues, Related to the STAR Methods (“Tiered strategy for estimating wildlife exposure under data-sparse conditions”).

Media/Products	Contents ($g\ g^{-1}$)				Reference
	Lipid (fat)	Non-lipid organics	Water	Others	
Blood	0.0023	0.1737	0.8090	0.0150	(Alencar & Rusig, 1983; Duarte et al., 1999)
Urine	0.0000	0.0000	0.9500	0.0500	(Gulhane et al., 2017b)
Bile	0.0056	0.0004	0.8940	0.1000	(Hertrampf & Piedad-Pascual, 2000)
Milk	0.0370	0.0840	0.8720	0.0070	(Li et al., 2022)
Liver	0.0360	0.2430	0.7080	0.0130	(*My Food Data - Free Nutrition Tools to Understand What You Eat*, n.d.-b)
Kidney	0.0310	0.1770	0.7790	0.0130	(*My Food Data - Free Nutrition Tools to Understand What You Eat*, n.d.-b)
Muscle	0.0280	0.2320	0.7310	0.0090	(WILLIAMS, 2007)
Fat	0.8000	0.0000	0.2000	0.0000	(Murphy, 1992)
Lung	0.0250	0.1620	0.7940	0.0098	(Li et al., 2022)
Mammary gland	0.1500	0.1300	0.7200	0.0000	(Li et al., 2022)

Table S10. Summary of physiological parameters of Sheep, Related to the STAR Methods (" Tiered strategy for estimating wildlife exposure under data-sparse conditions").

Physiological variables(cattle)	Symbol	Unit	Value	Reference
Food intake rate	IR_{Food}	kg day^{-1}	2.1	Estimated
Exhalation rate	$ER_{Exhalation}$	kg day^{-1}	25	(Gomes Silva et al., 2002)
Urine excretion rate	ER_{Urine}	kg day^{-1}	3	(Marsden et al., 2020)
Bile excretion rate	ER_{Bile}	kg day^{-1}	0.5	(Hofmann, 2007)
Milk excretion rate	ER_{Milk}	kg day^{-1}	1.5	(Castillo et al., 2009)
Body mass	---	kg	70	Estimated
Blood mass	M_{blood}	kg	3.3	(Lautz et al., 2020)
Liver mass	M_{liver}	kg	1	(Lautz et al., 2020)
Kidney mass	M_{kidney}	kg	0.2	(Lautz et al., 2020)
Lung mass	M_{lung}	kg	0.8	(Lautz et al., 2020)
Fat mass	M_{fat}	kg	13.4	(Lautz et al., 2020)
Muscle mass	M_{muscle}	kg	24.7	(Lautz et al., 2020)
Mammary gland mass	$M_{Mammary\ gland}$	kg	1.2	(Lautz et al., 2020)
Blood flow rate of liver	$Q_{Blood \Leftrightarrow Liver}$	kg day^{-1}	3788	(Lautz et al., 2020)
Blood flow rate of kidney	$Q_{Blood \Leftrightarrow Kidney}$	kg day^{-1}	1318	(Lautz et al., 2020)
Blood flow rate of lung	$Q_{Blood \Leftrightarrow Lung}$	kg day^{-1}	276	(Lautz et al., 2020)
Blood flow rate of fat	$Q_{Blood \Leftrightarrow Fat}$	kg day^{-1}	212	(Lautz et al., 2020)
Blood flow rate of muscle	$Q_{Blood \Leftrightarrow Muscle}$	kg day^{-1}	3060	(Lautz et al., 2020)
Blood flow rate of mammary gland	$Q_{Blood \Leftrightarrow Mammary\ gland}$	kg day^{-1}	682	(Lautz et al., 2020)

Table S11. Summary of nutritional composition of sheep tissues, Related to the STAR Methods ("Tiered strategy for estimating wildlife exposure under data-sparse conditions").

Media/Products	Contents (g g^{-1})				Reference
	Lipid (fat)	Non-lipid organics	Water	Others	
Blood	0.0023	0.1740	0.8090	0.0150	Estimated.
Urine	0.0000	0.0000	0.9500	0.0500	Estimated.
Bile	0.0056	0.0004	0.8940	0.1000	Estimated.
Milk	0.0370	0.0840	0.8720	0.0070	("Composition and Nutritional Value of Raw Milk," 2014)
Liver	0.0360	0.2430	0.7080	0.0130	(*Nutrition Facts Search Tool*, 2025)
Kidney	0.0310	0.1770	0.7790	0.0130	(*Nutrition Facts Search Tool*, 2025)
Muscle	0.0280	0.2320	0.7310	0.0090	(Williams, 2007)
Fat	0.8000	0.0000	0.2000	0.0000	(Murphy, 1992)
Lung	0.0250	0.1620	0.7940	0.0098	Estimated.
Mammary gland	0.1500	0.1300	0.7200	0.0000	Estimated.

Table S12. Summary of physiological parameters of Rabbit, Related to the STAR Methods (“ Tiered strategy for estimating wildlife exposure under data-sparse conditions”).

Physiological variables(rabbit)	Symbol	Unit	Value	Reference
Food intake rate	IR_{Food}	kg day^{-1}	0.15	(Houdebine & Fan, 2009)
Exhalation rate	$ER_{Exhalation}$	kg day^{-1}	1.05	Estimated from the tidal volume(0.61) and the breath per minute of cattle with the air density of 0.0012 kg L-1 (Houdebine & Fan, 2009)
Urine excretion rate	ER_{Urine}	kg day^{-1}	0.5	Physiological Features of Rabbits
Bile excretion rate	ER_{Bile}	kg day^{-1}	0.475	Estimated.(Bivolarski & Vachkova, 2014)
Body mass	---	kg	3.8	(Houdebine & Fan, 2009)
Blood mass	M_{blood}	kg	0.204	Estimated.(Prince, 1982)
Liver mass	M_{liver}	kg	0.13	Estimated (3.57%% of the body mass) (YUN Shi-feng et al., 2004)
Kidney mass	M_{kidney}	kg	0.023	Estimated (0.62%% of the body mass)(YUN Shi-feng et al., 2004)
Lung mass	M_{lung}	kg	0.012	Estimated (0.31%% of the body mass) (YUN Shi-feng et al., 2004)
Fat mass	M_{fat}	kg	0.23	Estimated (6.1%% of the body mass) (YUN Shi-feng et al., 2004)
Muscle mass	M_{muscle}	kg	2.11	Estimated (55.45% % of the body mass) 55.45% (YUN Shi-feng et al., 2004)
Blood flow rate of liver	$Q_{Blood \Leftrightarrow Liver}$	kg day^{-1}	28.51	(John et al., 1983; Reeves et al., 1988)
Blood flow rate of kidney	$Q_{Blood \Leftrightarrow Kidney}$	kg day^{-1}	107	(John et al., 1983; Reeves et al., 1988)
Blood flow rate of lung	$Q_{Blood \Leftrightarrow Lung}$	kg day^{-1}	12.1	(John et al., 1983; Reeves et al., 1988)
Blood flow rate of fat	$Q_{Blood \Leftrightarrow Fat}$	kg day^{-1}	64.8	(John et al., 1983; Reeves et al., 1988)
Blood flow rate of muscle	$Q_{Blood \Leftrightarrow Muscle}$	kg day^{-1}	191.5	(John et al., 1983; Reeves et al., 1988)

Table S13. Summary of nutritional composition of rabbit tissues, Related to the STAR Methods (" Tiered strategy for estimating wildlife exposure under data-sparse conditions").

Media/ Products	Contents (g g^{-1}) Lipid (fat)	Non-lipid organics	Water	Others	Reference
Blood	0.0070	0.0670	0.9260	0.0000	(Mavroudis et al., 2018)
Urine	0.0000	0.0000	0.9500	0.0500	Estimated.
Bile	0.0056	0.0004	0.8940	0.1000	Estimated.
Liver	0.0690	0.1840	0.7470	0.0000	(Mavroudis et al., 2018)
Kidney	0.0520	0.1710	0.7740	0.0030	(Mavroudis et al., 2018)
Muscle	0.0130	0.1770	0.8100	0.0000	(Mavroudis et al., 2018)
Fat	0.8000	0.0500	0.1500	0.0000	(Mavroudis et al., 2018)
Lung	0.0100	0.1830	0.8070	0.0000	(Mavroudis et al., 2018)

Table S14. Summary of physiological parameters of Fox, Related to the STAR Methods ("Tiered strategy for estimating wildlife exposure under data-sparse conditions").

Physiological variables	Symbol	Unit	Value	Reference
Food intake rate	$IR_{n \rightarrow n+1,oral}$	kg day^{-1}	2.50	Estimated.
Exhalation rate	$ER_{Exhalation}$	kg day^{-1}	1.98	Estimated.
Urine excretion rate	ER_{Urine}	kg day^{-1}	0.20	Estimated.
Bile excretion rate	ER_{Bile}	kg day^{-1}	0.09	Estimated.
Body mass	---	kg	5.75	Estimated.
Blood mass	M_{blood}	kg	0.46	(Du Dot et al., 2009)
Liver mass	M_{liver}	kg	0.21	(Wei et al., 2020)
Kidney mass	M_{kidney}	kg	0.03	(Wei et al., 2020)
Lung mass	M_{lung}	kg	0.04	(Wei et al., 2020)
Fat mass	M_{fat}	kg	0.58	(Paolo CAVALLINI, 1997)
Muscle mass	M_{muscle}	kg	2.30	(Paolo CAVALLINI, 1997)
Blood flow rate of liver	$Q_{Blood \Leftrightarrow Liver}$	kg day^{-1}	2138.40	Estimated.
Blood flow rate of kidney	$Q_{Blood \Leftrightarrow Kidney}$	kg day^{-1}	1700.80	(Valitskaya et al., 1988)
Blood flow rate of lung	$Q_{Blood \Leftrightarrow Lung}$	kg day^{-1}	2376.00	(Valitskaya et al., 1988)
Blood flow rate of fat	$Q_{Blood \Leftrightarrow Fat}$	kg day^{-1}	665.28	(Longworth et al., 1989; Valitskaya et al., 1988)
Blood flow rate of muscle	$Q_{Blood \Leftrightarrow Muscle}$	kg day^{-1}	1663.20	(Valitskaya et al., 1988)

Table S15. Summary of nutritional composition of fox tissues, Related to the STAR Methods (“ Tiered strategy for estimating wildlife exposure under data-sparse conditions”).

Media/Products	Contents ($g\ g^{-1}$)				References
	Lipid (fat)	Non-lipid organics	Water	Others	
Blood	0.0020	0.074	0.9202	0.0038	(Xiao et al., 1998)
Urine	0	0	0.95	0.05	Estimated.
Bile	0.0175	0.035	0.90	0.0475	Estimated.
Liver	0.052	0.15	0.75	0.028	(Kelly & Gobas, 2001)
Lung	0.04	0.18	0.77	0.01	Estimated.
Kidney	0.03	0.165	0.792	0.013	(*My Food Data - Free Nutrition Tools to Understand What You Eat*, n.d.-a)
Fat	0.8793	0	0.1207	0	(Kelly & Gobas, 2001)
Muscle	0.029	0.2259	0.74	0.00512	(Fan et al., 2015; Kelly & Gobas, 2001)

Table S16. Summary of physiological parameters of wolf, Related to the STAR Methods (“ Tiered strategy for estimating wildlife exposure under data-sparse conditions”).

Symbol	Variables	Value	Unit	Note
$IR_{deer\rightarrow wolf,Oral}$	Daily deer intake mass by wolf	1.5	mg kg^{-1}	On average, a wolf consumes about 3 kilograms of meat per day, assuming that deer make up 50% of the wolf's diet.
$ER_{Bile,Wolf}$	Biliary excretion rate	0.3	kg day^{-1}	(Gobas et al., 2003)
$ER_{Exhalation,Wolf}$	Expiratory rate	18.06	kg day^{-1}	One cubic metre of air has a mass of 1.29 kg. (Arctic Food Web)(Kelly et al., 2007)
$ER_{Urine,Wolf}$	Urinary excretion rate	1.0	kg day^{-1}	Estimated (Gobas et al., 2003)
$M_{Body,Wolf}$	Wolf body weight	90	kg	Estimated (Kelly et al., 2007)
$M_{Blood,Wolf}$	Wolf blood quality	7.2	kg	Estimated (8%% of the body mass).(Müller et al., 2011)
$M_{Liver,Wolf}$	Wolf liver quality	2.88	kg	Estimated (3.2%% of the body mass).(Müller et al., 2011)3.2%
$M_{Lung,Wolf}$	Wolf lung quality	1.26	kg	Estimated (1.4%% of the body mass).(Müller et al., 2011)1.4%
$M_{Kidney,Wolf}$	Wolf kidney quality	0.63	kg	Estimated (0.7%% of the body mass).(Müller et al., 2011)0.7%
$M_{Fat,Wolf}$	Wolf fat quality	10.8	kg	Estimated (12%% of the body mass).(Morris et al., 2018)
$M_{Muscle,Wolf}$	Wolf muscle quality	45.99	kg	Estimated (51.1%% of the body mass).(Müller et al., 2011)
$Q_{B\Leftrightarrow Liver,Wolf}$	Blood flow rate of compartment liver	3199	kg day^{-1}	Cardiac output 7830 ml/per minutes(7.48kg) Estimated from dog 29.7% (Brown et al., 1997)
$Q_{B\Leftrightarrow Lung,Wolf}$	Blood flow rate of compartment lung	948	kg day^{-1}	Estimated from dog 8.8% (Brown et al., 1997)
$Q_{B\Leftrightarrow Kidney,Wolf}$	Blood flow rate of compartment kidney	1863	kg day^{-1}	Estimated from dog 17.3% (Brown et al., 1997)
$Q_{B\Leftrightarrow Fat,Wolf}$	Blood flow rate of compartment fat	560	kg day^{-1}	Estimated from human 5.2% (Brown et al., 1997)
$Q_{B\Leftrightarrow Muscle,Wolf}$	Blood flow rate of compartment muscle	2337	kg day^{-1}	Estimated from dog 21.7% (Brown et al., 1997)

Table S17. Summary of nutritional composition of wolf tissues, Related to the STAR Methods (“ Tiered strategy for estimating wildlife exposure under data-sparse conditions”).

Media/Products	Contents (g g^{-1})				References
	Lipid (fat)	Non-lipid organics	Water	Others	
Blood	0.0020	0.074	0.9202	0.0038	(Xiao et al., 1998)
Urine	0	0	0.95	0.05	Estimated.
Bile	0.0175	0.035	0.90	0.0475	Estimated.
Liver	0.052	0.15	0.75	0.028	(Kelly & Gobas, 2001)
Lung	0.04	0.18	0.77	0.01	Estimated.
Kidney	0.03	0.165	0.792	0.013	(*My Food Data - Free Nutrition Tools to Understand What You Eat*, n.d.-a)
Fat	0.8793	0	0.1207	0	(Kelly & Gobas, 2001)
Muscle	0.029	0.2259	0.74	0.00512	(Fan et al., 2015; Kelly & Gobas, 2001)

Table S18. Summary of physiological parameters of Lion, Related to the STAR Methods (“ Tiered strategy for estimating wildlife exposure under data-sparse conditions”).

Physiological variables(wolf)	Symbol	Unit	Value	Reference
Food intake rate	$IR_{n \rightarrow n+1,oral}$	kg day^{-1}	6.5	(Jackie A. Rapson & Ric T.F. Bernard, 2007)
Exhalation rate	$ER_{Exhalation}$	kg day^{-1}	39.4	(Donaldson et al., 2023)
Urine excretion rate	ER_{Urine}	kg day^{-1}	5.28	(Smith et al., 2006)
Bile excretion rate	ER_{Bile}	kg day^{-1}	3.55	Estimated.
Body mass	---	kg	176	(Davis, 1962)
Blood mass	M_{blood}	kg	13.2	(Davis, 1962)
Liver mass	M_{liver}	kg	4.88	(Davis, 1962)
Kidney mass	M_{kidney}	kg	1.25	(Davis, 1962)
Lung mass	M_{lung}	kg	3.52	(Davis, 1962)
Fat mass	M_{fat}	kg	5.47	(Davis, 1962)
Muscle mass	M_{muscle}	kg	98.7	(Davis, 1962)
Blood flow rate of liver	$Q_{Blood \Leftrightarrow Liver}$	kg day^{-1}	24494.4	Estimated.(Van Valkenburgh et al., 2004)
Blood flow rate of kidney	$Q_{Blood \Leftrightarrow Kidney}$	kg day^{-1}	17962.56	Estimated.(Van Valkenburgh et al., 2004)
Blood flow rate of lung	$Q_{Blood \Leftrightarrow Lung}$	kg day^{-1}	32659.2	Estimated.(Van Valkenburgh et al., 2004)
Blood flow rate of fat	$Q_{Blood \Leftrightarrow Fat}$	kg day^{-1}	3265.92	Estimated.(Van Valkenburgh et al., 2004)
Blood flow rate of muscle	$Q_{Blood \Leftrightarrow Muscle}$	kg day^{-1}	35380.8	Estimated.(Van Valkenburgh et al., 2004)

Table S19. Summary of nutritional composition of lion tissues, Related to the STAR Methods ("Tiered strategy for estimating wildlife exposure under data-sparse conditions").

Media/Products	Contents ($g\ g^{-1}$)				Reference
	Lipid (fat)	Non-lipid organics	Water	Others	
Blood	0.0055	0.18	0.8	0.0145	Estimated.
Urine	0.001	0.045	0.95	0.004	Estimated.
Bile	0.0175	0.035	0.9	0.0475	Estimated.
Liver	0.043	0.25	0.69	0.017	Estimated.
Kidney	0.02	0.21	0.77	0	Estimated. (Kelly & Gobas, 2001)
Muscle	0.025	0.25	0.715	0.01	Estimated.
Fat	0.915	0.0125	0.07	0.0025	Estimated.(Kelly & Gobas, 2001)
Lung	0.029	0.2259	0.74	0.0051	Estimated.(Fan et al., 2015; Kelly & Gobas, 2001)

Table S20. Summary of physiological parameters of Wild boar, Related to the STAR Methods (" Tiered strategy for estimating wildlife exposure under data-sparse conditions").

Physiological variables	Symbol	Unit	Value	Reference
Food intake rate	$IR_{n\rightarrow n+1,oral}$	kg day^{-1}	3.00	Estimated.
Exhalation rate	$ER_{Exhalation}$	kg day^{-1}	136.00	Estimated.
Urine excretion rate	ER_{Urine}	kg day^{-1}	4.00	Estimated.
Bile excretion rate	ER_{Bile}	kg day^{-1}	2.00	Estimated.
Body mass	---	kg	100.00	(Lautz et al., 2020)
Blood mass	M_{blood}	kg	3.00	(Lautz et al., 2020)
Liver mass	M_{liver}	kg	1.70	(Lautz et al., 2020)
Kidney mass	M_{kidney}	kg	0.85	(Lautz et al., 2020)
Lung mass	M_{lung}	kg	0.80	(Lautz et al., 2020)
Fat mass	M_{fat}	kg	17.60	(Lautz et al., 2020)
Muscle mass	M_{muscle}	kg	60.00	(Lautz et al., 2020)
Blood flow rate of liver	$Q_{Blood\Leftrightarrow Liver}$	kg day^{-1}	7819.20	(Upton, 2008; Yang & Lin, 1997)
Blood flow rate of kidney	$Q_{Blood\Leftrightarrow Kidney}$	kg day^{-1}	12435.80	(Upton, 2008; Yang & Lin, 1997)
Blood flow rate of lung	$Q_{Blood\Leftrightarrow Lung}$	kg day^{-1}	15504.50	(Upton, 2008; Yang & Lin, 1997)
Blood flow rate of fat	$Q_{Blood\Leftrightarrow Fat}$	kg day^{-1}	1706.40	(Upton, 2008; Yang & Lin, 1997)
Blood flow rate of muscle	$Q_{Blood\Leftrightarrow Muscle}$	kg day^{-1}	15854.40	(Upton, 2008; Yang & Lin, 1997)

Table S21. Summary of nutritional composition of wild boar tissues, Related to the STAR Methods ("Tiered strategy for estimating wildlife exposure under data-sparse conditions").

Media/Products	Contents ($g\ g^{-1}$)				Reference
	Lipid (fat)	Non-lipid organics	Water	Others	
Blood	0.0030	0.1310	0.8580	0.0080	(China CDC, 2025)
Urine	0.0000	0.0000	0.9500	0.0500	Estimated.
Bile	0.0018	0.0350	0.9000	0.0475	Estimated.
Liver	0.0350	0.2430	0.7070	0.0150	(China CDC, 2025)
Kidney	0.0320	0.1680	0.7880	0.0120	(China CDC, 2025)
Muscle	0.0620	0.2180	0.7100	0.0100	(China CDC, 2025)
Fat	0.8860	0.0240	0.0880	0.0020	(China CDC, 2025)
Lung	0.0390	0.1230	0.8310	0.0070	(China CDC, 2025)

Table S22. Summary of physiological parameters of Giant panda, related to the STAR Methods (" Tiered strategy for estimating wildlife exposure under data-sparse conditions").

Physiological variables	Symbol	Unit	Value	Reference
Food intake rate	$IR_{n\rightarrow n+1,oral}$	kg day^{-1}	15.00	Estimated.
Exhalation rate	$ER_{Exhalation}$	kg day^{-1}	136.00	Estimated.
Urine excretion rate	ER_{Urine}	kg day^{-1}	2.20	(Huang et al., 2016)
Bile excretion rate	ER_{Bile}	kg day^{-1}	1.00	(Peng Yingjun., 1995)
Body mass	---	kg	100.00	(Lai Dehua et al., 1990)
Blood mass	M_{blood}	kg	8.00	(Dong Quan et al., 1991; Lai Dehua et al., 1990)
Liver mass	M_{liver}	kg	1.79	(Lai Dehua et al., 1990)
Kidney mass	M_{kidney}	kg	0.36	(Lai Dehua et al., 1990)
Lung mass	M_{lung}	kg	1.85	(Lai Dehua et al., 1990)
Fat mass	M_{fat}	kg	10.00	(Lai Dehua et al., 1990)
Muscle mass	M_{muscle}	kg	37.50	(Lai Dehua et al., 1990)
Blood flow rate of liver	$Q_{Blood\Leftrightarrow Liver}$	kg day^{-1}	10530.00	Estimated.(Laske et al., 2017)
Blood flow rate of kidney	$Q_{Blood\Leftrightarrow Kidney}$	kg day^{-1}	8424.00	(Laske et al., 2017)
Blood flow rate of lung	$Q_{Blood\Leftrightarrow Lung}$	kg day^{-1}	11700.00	Estimated.(Laske et al., 2017)
Blood flow rate of fat	$Q_{Blood\Leftrightarrow Fat}$	kg day^{-1}	3276.00	Estimated.(Laske et al., 2017)
Blood flow rate of muscle	$Q_{Blood\Leftrightarrow Muscle}$	kg day^{-1}	8190.00	Estimated.(Laske et al., 2017)

Table S23. Summary of nutritional composition of giant panda tissues, related to the STAR Methods (“Tiered strategy for estimating wildlife exposure under data-sparse conditions”).

Media/Products	Contents (g g^{-1})				References
	Lipid (fat)	Non-lipid organics	Water	Others	
Blood	0.0020	0.074	0.9202	0.0038	(Xiao et al., 1998)
Urine	0	0	0.95	0.05	Estimated.
Bile	0.0175	0.035	0.90	0.0475	Estimated.
Liver	0.052	0.15	0.75	0.028	(Kelly & Gobas, 2001)
Lung	0.04	0.18	0.77	0.01	Estimated.
Kidney	0.03	0.165	0.792	0.013	(*My Food Data - Free Nutrition Tools to Understand What You Eat*, n.d.-a)
Fat	0.8793	0	0.1207	0	(Kelly & Gobas, 2001)
Muscle	0.029	0.2259	0.74	0.00512	(Fan et al., 2015; Kelly & Gobas, 2001)

Table S24. The physicochemical parameters of the chemical substances involved in this research, Related to the STAR Methods (“Exposure and ecological risk assessments”).

**Chemical**	**CAS.No**	**Biotransformation half-life(day)**	**Log Kow**	**Log Kaw**	**Log K_{OC}**
o.p’-DDE (2,4'-DDE）	3424-82-6	122.8	5.87E+00	-2.72E+00	5.41E+00
p.p’-DDE (4,4'-DDE)	72-55-9	252	6.51E+00	-2.77E+00	6.05E+00
o.p’-DDD (2,4'-DDD）	53-19-0	53	5.87E+00	-3.48E+00	5.41E+00
p.p’-DDD (4,4'-DDD)	72-54-8	58.8	6.02E+00	-3.57E+00	5.56E+00
o.p’-DDT(2,4'-DDT)	789-02-6	149	6.79E+00	-3.52E+00	6.33E+00
p.p’-DDT(4,4'-DDT)	50-29-3	161	6.91E+00	-3.47E+00	6.45E+00
α-HCH	319-84-6	17	3.80E+00	-3.68E+00	3.34E+00
β-HCH	319-85-7	17	3.78E+00	-3.68E+00	3.32E+00
γ-HCH(Lindane)	58-89-9	17	3.72E+00	-3.68E+00	3.26E+00
σ-HCH	27176-87-0	1.32	4.78E+00	-8.70E+00	4.32E+00
HCB	118-74-1	21.5	5.73E+00	-1.16E+00	5.27E+00
trans-Chlordane (TC)	5103-74-2	129	6.22E+00	-2.70E+00	5.76E+00
cis-Chlordane (CC)	5103-71-9	129	6.10E+00	-2.70E+00	5.64E+00
α-Endosulfan	959-98-8	5.39	3.83E+00	-2.58E+00	3.37E+00
β-Endosulfan	33213-65-9	5.39	3.83E+00	-2.58E+00	3.37E+00
Aldrin	309-00-2	100	6.50E+00	-2.75E+00	6.04E+00
Dieldrin	60-57-1	103	5.40E+00	-3.39E+00	4.94E+00
Heptachlor	76-44-8	50.1	6.10E+00	-1.92E+00	5.64E+00
Heptachlor-epoxide	1024-57-3	33.7	4.98E+00	-3.07E+00	4.52E+00
Endrin	72-20-8	103	5.20E+00	-3.39E+00	4.74E+00
Mirex	2385-85-5	109	6.89E+00	-1.48E+00	6.43E+00

Methoxychlor	72-43-5	18.4	5.08E+00	-5.08E+00	4.62E+00
Metolachlor	51218-45-2	0.184	3.13E+00	-6.07E+00	2.67E+00
Deltamethrin	52918-63-5	0.544	6.20E+00	-3.69E+00	5.74E+00
Acetochlor	34256-82-1	0.289	4.14E+00	-6.53E+00	3.68E+00
Atrazine	001912-24-9	0.089	2.61E+00	-6.68E+00	2.15E+00
chlorpyrifos（CPF)	002921-88-2	5.92	4.96E+00	-3.32E+00	4.50E+00
dicofol (DCF)	115-32-2	37.4	5.02E+00	-6.40E+00	4.56E+00
Imidacloprid	138261-41-3	0.013	5.70E-01	-1.06E+01	1.14E-01
Methamidophos	10265-92-6	0.022	-8.00E-01	-8.26E+00	-1.26E+00
Malathion	121-75-5	0.007	2.36E+00	-6.70E+00	1.90E+00
Glyphosate	1071-83-6	0.0025	-3.40E+00	-1.44E+01	-3.86E+00
trifluralin (TFL)	1582-09-8	0.561	5.27E+00	-2.56E+00	4.81E+00
chlorothalonil (CTN)	1897-45-6	1.29	2.90E+00	-5.05E+00	2.44E+00
cypermethrin (CMT)	52315-07-8	2.32	6.60E+00	-5.14E+00	6.14E+00
Pentachlorobenzene	608-93-5	13.7	5.18E+00	-1.52E+00	4.72E+00
Pentachloroanisole	1825-21-4	69	5.45E+00	-7.20E-01	4.99E+00
Octachlorostyrene	29082-74-4	212	7.14E+00	-2.28E+00	6.68E+00

303

Table S25. Summary of Pollution Scores from Different Sampling Sources (Soil, Plants, and Mammals), Related to the STAR Methods (“Exposure and ecological risk assessments”).

Type	Sample of Pesticides	Sample of Country	Sample of Articles	Median Concentration (IQR)	Median Score (IQR)
Carnivores Liver	727	8	15	284 (113—2720)	-0.21 (-0.72—0.38)
Herbivores Liver	445	7	14	590 (17.8—1889)	-0.60 (-1.74—0.13)
Omnivores Liver	407	6	9	117 (67.4—225.6)	-0.81 (-1.07— -0.52)
Soil	2436	13	46	6.60 (1.96—36.5)	-3.24 (-3.83— -2.52)
Wild Plant	1016	10	15	340.36 (7.4—20740)	-2.06 (-3.08— -1.02)

Table S26. Statistical Distribution of Animal-Derived Exposure and Ecological Risk Assessment (ECR, HQ) Data, Related to the Result ("Wildlife exposure and risk estimation").

Country	Species	Data Source	Median Log ECR (IQR)	Log ECR Min-Max	Median Log HQ (IQR)	Log HQ Min-Max
Norway	Fox	Animal	**-3.74(-4.03 — -3.39)**	**-5.96 — -3.20**	**0.77(0.71—0.83)**	**0.63—0.88**
Spain	Fox	Animal	-5.67(-5.81 — -4.85)	-7.00 — -4.64	-0.81(-1.11 — -0.77)	-3.02 — -0.74
Croatia	Wolf	Animal	-6.58 (-9.04 — -6.24)	-10.03 — -6.16	-2.34 (-2.64 — -2.13)	-5.32 — -1.98
South Africa	Lion	Animal	**-2.66(-2.70 — -2.32)**	**-2.82 — -1.78**	1.97 (1.93 — 2.01)	1.88 — 2.04
Mexico	Cattle	Animal	**-2.87(-3.02 — -2.07)**	**-3.09 — -1.78**	**2.74(2.72 — 2.76)**	**2.69 — 2.78**
Denmark	Caribou	Animal	-4.61(-4.79 — -4.49)	-5.07 — -4.40	-1.82(-2.97 — -0.77)	-2.97 — 0.26
Canada	Moose	Animal	-7.44(-7.83 — -5.58)	-8.52 — -5.17	-1.77(-2.34 — -0.97)	-2.78 — 0.32
Columbia	Chiroptera	Animal	-3.74(-3.93 — -3.51)	-4.25 — -2.42	-0.29(-0.50 — 0.10)	-1.13 — 0.35
Jordan	Sheep	Animal	**-2.18(-2.93 — -1.70)**	**-3.50 — -1.61**	**2.67(2.25 — 3.47)**	**2.08 — 3.96**
China	Panda	Animal	**-1.19(-1.29 — -0.83)**	**-1.94 — -0.23**	**3.73(3.43 — 3.91)**	**0.98 — 4.03**
Poland	Wild boar	Animal	**0.34(0.23 — 0.35)**	**0.07 — 0.37**	**6.49(6.21 — 6.66)**	**5.22 — 6.78**
Vojvodina	Wild boar	Animal	**0.22(-0.13 — 0.32)**	**-0.86 — 0.64**	**4.99(4.17 — 5.80)**	**3.10 — 6.49**

Supplemental Method

Methods 1: Literature Search Strategy

We conducted a systematic literature search using the Web of Science database for publications from January 1, 2000, to December 31, 2024. Keyword combinations were designed using the AND operator, as detailed below.

Wild mammals: Search terms included “wild herbivores,” “wild omnivores,” “wild mammals,” or “wild animals,” combined with pesticide-related terms (“pesticide,” “insecticide,” “herbicide,” or “fungicide”) and contamination indicators (“residuals,” “pollution,” or “concentration”).

Soils: Search terms for soil-related research included “ecological soil,” “highland soil,” “mountain soil,” “forest soil,” “grassland soil,” “hilly soil,” or “plain soil,” combined with pesticide-related and contamination-related keywords.

Wild plants: Search terms for plant-related research included “wild plants,” “vegetation,” “shrubs,” “wildflowers,” “weeds,” or “trees,” combined with pesticide-related and concentration descriptors.

All searches were limited to studies reporting empirical measurements. Review articles, meta-analyses, modeling studies, and secondary analyses were excluded. Only samples from natural, non-degraded, and non-urbanized environments were retained.

Method S2. Soil-plant transfer model

$BAF_{plant}^{H}(T_{Air}, RH_{Air})$is derived from the pesticide mass balance of herbaceous plant leaves based on a single-compartment model. Therefore, when $C_W \leq S$ (T_S) and there are steady-state conditions, the pesticide BAF in the leaves of herbaceous plants can be expressed as follows (The meaning and interpretation of specific values are provided in Table S1)：

$$BAF_{plant}^{H}(T_{Air}, RH_{Air}) = \frac{Q^{H}(T_{Air}, RH_{Air}) \times \frac{1}{k_d}}{A_L^H \cdot g \cdot \frac{1000}{K_{LA}} + \lambda_L^H \cdot M_L^H} \quad \text{Equation S1}$$

The transpiration rate of herbaceous plant leaves is related to the surrounding atmospheric conditions, which can be expressed using $Q^{H}(T_{Air}, RH_{Air})$. The specific formula is:

$$Q^{H}(T_{Air}, RH_{Air}) = \frac{\rho_{Air} c_p LAI}{\rho_{Water} \Delta H_{Vap} \gamma} \left(\frac{s\Delta T + VPD}{R_S + R_{Air}}\right)\left(\frac{86400s}{d}\right) \quad \text{Equation S2}$$

The k_d ($\frac{mg \cdot kg^{-1}}{mg \cdot L^{-1}}$)value is used to estimate pesticide concentrations in water; this value represents the partition coefficient between dry soil and water, as well as the initial concentration of pesticides in dry soil.

$$k_d = K_{oc} \times f_{oc} \quad \text{Equation S3}$$

In Eq. (4), is estimated from the leaf water ($L \cdot kg^{-1}$) and lipid ($kg \cdot kg^{-1}$) contents, the octanol-water partition coefficient (K_{OW}), and the density of octanol ($kg \cdot L^{-1}$), and cc is the corrected coefficient .

$$K_{LA} = \frac{W_L + \frac{L_L(K_{ow})^{cc}}{\rho_{oc}}}{K_{AW}} \qquad \text{Equation S4}$$

s ($Pa \cdot K^{-1}$) denotes the slope between saturated vapour pressure (Pa) and temperature (K) for air.

$$s = \frac{2.50 \times 10^6 \times e^{\frac{17.2(T_{Air} - 273.15)}{T_{Air} - 35.85}}}{(T_{Air} - 35.85)^2} \qquad \text{Equation S5}$$

VPD (Pa) denotes the vapour pressure deficit, expressed by Equation (6). (K) represents the temperature difference between the leaf and the air, assumed to be due to the continuous transport of water. The difference between the soil and the air is expressed by Equation (7).

$$VPD = 610.78(1 - \frac{RH_{Air}}{100})\, e^{\frac{17.2(T_{Air} - 273.15)}{T_{Air} - 35.85}} \qquad \text{Equation S6}$$

$$\Delta T = T_{leaf} - T_{Air} = 31.145 - 0.1T_{Air} \qquad \text{Equation S7}$$

Following simplification, the BAF for herbaceous plants was obtained.

$$BAF^{H}_{plant}(T_{Air}, RH_{Air}) = \frac{\frac{\rho_{Air} c_p LAI}{\rho_{Water} \Delta H_{Vap} \gamma} \left(\frac{s\Delta T + VPD}{R_S + R_{Air}}\right) \left(\frac{86400s}{d}\right) \left(\frac{1}{k_d}\right)}{g(\frac{1000L}{m^3}) \left(\frac{1}{K_{LA}}\right) + \lambda^{H} \cdot LMA^{H}_{L}} \qquad \text{Equation S8}$$

References

Adamczewski, J. Z., Flood, P. F., & Gunn, A. (1995). Body composition of muskoxen (Ovibos moschatus) and its estimation from condition index and mass measurements. *Canadian Journal of Zoology*, *73*(11), 2021–2034. https://doi.org/10.1139/Z95-238

Alencar, F. A., & Rusig, O. (1983). *Estudos da recuperação das proteinas do plasma bovino por complexação com fosfatos e sua utilização em produtos carneos*.

Ashara, K. C., & Shah, K. (2016). *Cow's Urine: An Incredible Aqueous Phase*.

Bivolarski, B. L., & Vachkova, E. G. (2014). Morphological and functional events associated to weaning in rabbits. *Journal of Animal Physiology and Animal Nutrition*, *98*(1), 9–18. https://doi.org/10.1111/jpn.12058

Brown, R. P., Delp, M. D., Lindstedt, S. L., Rhomberg, L. R., & Beliles, R. P. (1997). Physiological Parameter Values for Physiologically Based Pharmacokinetic Models. *Toxicology and Industrial Health*, *13*(4), 407–484. https://doi.org/10.1177/074823379701300401

Castillo, V., Such, X., Caja, G., Casals, R., Salama, A. A. K., & Albanell, E. (2009). Long- and short-term effects of omitting two weekend milkings on the lactational performance and mammary tight junction permeability of dairy ewes. *Journal of Dairy Science*, *92*(8), 3684–3695. https://doi.org/10.3168/jds.2008-1937

China CDC. (2025). *Pre-packaged Food Nutrition Labelling Data Query System*. https://nlc.chinanutri.cn/

Composition and nutritional value of raw milk. (2014). *Issues in Biological Sciences and Pharmaceutical Research, Vol , Page - , 2014*. https://journalissues.org/ibspr/abstract/guetouache-et-al-december-2014/

Davis, D. D. (1962). Allometric Relationships in Lions vs. Domestic Cats. *Evolution*, *16*(4), 505. https://doi.org/10.2307/2406182

DelGiudice, G. D., Asleson, M. A., Hellgren, E. C., & Varner, L. W. (1995). Twenty-four-hour urinary creatinine and urea nitrogen excretion in male white-tailed deer. *Canadian Journal of Zoology*, *73*(3), 493–501. https://doi.org/10.1139/z95-056

Donald G. Stevens. (1981). A Model of Respiratory Vapor Loss in Holstein Dairy Cattle. *Transactions of the ASAE*, *24*(1), 0151–0153. https://doi.org/10.13031/2013.34215

Donaldson, A. C., Meyer, L. C. R., Fuller, A., & Buss, P. E. (2023). Comparison of the cardiovascular effects of immobilization with three different drug combinations in free-ranging African lions. *Conservation Physiology*, *11*(1). https://doi.org/10.1093/conphys/coac077

Dong Quan, Long Zhi, Ji Meirong, Zhang Anju, Ye Zhiyong, Tian Huajian, Xiang Peilun, & Huang Hua. (1991). Determination of Blood Components in Giant Pandas (Chinese). *Acta Zoologica Sinica*, *1*(12), 64–75.

Du Dot, T. J., Rosen, D. A. S., & Trites, A. W. (2009). Energy reallocation during and after periods of nutritional stress in steller sea lions: Low-quality diet reduces capacity for physiological adjustments. *Physiological and Biochemical Zoology*, *82*(5), 516–530. https://doi.org/10.1086/603637

Duarte, R. T., Carvalho Simões, M. C., & Sgarbieri, V. C. (1999). Bovine Blood Components: Fractionation, Composition, and Nutritive Value. *Journal of Agricultural and Food Chemistry*, *47*(1), 231–236. https://doi.org/10.1021/jf9806255

Fan, L., Zhang, Z., & Zhang Li. (2015). Analysis of nutritional components and nutritional evaluation of Huajiang dog meat. *Meat Industry*.

Gerhart, K. L., White, R. G., Cameron, R. D., & Russell, D. E. (1996). Body composition and nutrient reserves of arctic caribou. *Canadian Journal of Zoology*, *74*(1), 136–146. https://doi.org/10.1139/z96-018

Gobas, F. A. P. C., Kelly, B. C., & Arnot, J. A. (2003). Quantitative Structure Activity Relationships for Predicting the Bioaccumulation of POPs in Terrestrial Food-Webs. *QSAR & Combinatorial Science*, *22*(3), 329–336. https://doi.org/10.1002/qsar.200390022

Gomes da Silva, R., LaScala, N., Lima Filho, A., & Catharin, M. (2002). Respiratory heat loss in the sheep: a comprehensive model. *International Journal of Biometeorology*, *46*(3), 136–140. https://doi.org/10.1007/s00484-002-0128-0

Gulhane, H., Nakanekar, A., Mahakal, N., Bhople, S., & Salunke, A. (2017a). GOMUTRA (COW URINE): A MULTIDIMENSIONAL DRUG REVIEW ARTICLE. *International Journal of Research in Ayurveda & Pharmacy*, *8*(5), 1–6. https://doi.org/10.7897/2277-4343.085231

Gulhane, H., Nakanekar, A., Mahakal, N., Bhople, S., & Salunke, A. (2017b). GOMUTRA (COW URINE): A MULTIDIMENSIONAL DRUG REVIEW ARTICLE. *International Journal of Research in Ayurveda & Pharmacy*, *8*(5), 1–6. https://doi.org/10.7897/2277-4343.085231

Hertrampf, J. W., & Piedad-Pascual, F. (2000). *Handbook on Ingredients for Aquaculture Feeds*. Springer Netherlands. https://doi.org/10.1007/978-94-011-4018-8

Hofmann, A. F. (2007). Biliary secretion and excretion in health and disease: Current concepts. *Annals of Hepatology*, *6*(1), 15–27. https://doi.org/10.1016/S1665-2681(19)31949-0

Houdebine, L. M., & Fan, J. (2009). Rabbit biotechnology: Rabbit genomics, transgenesis, cloning and models. *Rabbit Biotechnology: Rabbit Genomics, Transgenesis, Cloning and Models*, 1–132. https://doi.org/10.1007/978-90-481-2227-1

Huang, H., Yie, S., Liu, Y., Wang, C., Cai, Z., Zhang, W., Lan, J., Huang, X., Luo, L., Cai, K., Hou, R., & Zhang, Z. (2016). Dietary resources shape the adaptive changes of cyanide detoxification function in giant panda (Ailuropoda melanoleuca). *Scientific Reports 2016 6:1*, *6*(1), 34700-. https://doi.org/10.1038/srep34700

Hugh-Jones, P., Barter, C. E., Hime, J. M., & Rusbridge, M. M. (1978). Dead space and tidal volume of the giraffe compared with some other mammals. *Respiration Physiology*, *35*(1), 53–58. https://doi.org/10.1016/0034-5687(78)90040-3

Jackie A. Rapson, & Ric T.F. Bernard. (2007, October 1). *Interpreting the diet of lions (Panthera leo); a comparison of various methods of analysis* . South African Journal of Wildlife Research. https://journals.co.za/doi/abs/10.10520/EJC117267

Jensen, W. F., Smith, J. R., Jr., J. J. M., (Deceased), J. V. M., & (Retired), R. E. J. (2013). MASS, MORPHOLOGY, AND GROWTH RATES OF MOOSE IN NORTH DAKOTA. *Alces*, *49*, 1–15. https://alcesjournal.org/index.php/alces/article/view/107

John, E., McDevitt, M., & Cassady, G. (1983). Cardiac Output and Organ Blood Flow in Young Rabbits during Intermittent Positive-Pressure Ventilation. *Neonatology*, *44*(1), 58–64. https://doi.org/10.1159/000241696

Jürgens, K. D., Bartels, H., & Bartels, R. (1981). Blood oxygen transport and organ weights of small bats and small non-flying mammals. *Respiration Physiology*, *45*(3), 243–260. https://doi.org/10.1016/0034-5687(81)90009-8

Kelly, B. C., & Gobas, F. A. P. C. (2001). Bioaccumulation of Persistent Organic Pollutants in Lichen−Caribou−Wolf Food Chains of Canada's Central and Western Arctic. *Environmental Science & Technology*, *35*(2), 325–334. https://doi.org/10.1021/es0011966

Kelly, B. C., Ikonomou, M. G., Blair, J. D., Morin, A. E., & Gobas, F. A. P. C. (2007). Food Web–Specific Biomagnification of Persistent Organic Pollutants. *Science*, *317*(5835), 236–239. https://doi.org/10.1126/science.1138275

Lai Dehua, Cao Junming, & Wang Weiyun. (1990). Measurement and Preliminary Analysis of Selected Organs in Four Giant Pandas (Chinese)). *Journal of Southwest University of Science and Technology (Philosophy and Social Sciences Edition)*, *01*, 50–53.

Laske, T. G., Iaizzo, P. A., & Garshelis, D. L. (2017). Six Years in the Life of a Mother Bear - The Longest Continuous Heart Rate Recordings from a Free-Ranging Mammal. *Scientific Reports*, *7*(1), 40732. https://doi.org/10.1038/srep40732

Lautz, L. S., Dorne, J. L. C. M., Oldenkamp, R., Hendriks, A. J., & Ragas, A. M. J. (2020). Generic physiologically based kinetic modelling for farm animals: Part I. Data collection of physiological parameters in swine, cattle and sheep. *Toxicology Letters*, *319*, 95–101. https://doi.org/10.1016/j.toxlet.2019.10.021

Li, Z., Xiong, J., & Fantke, P. (2022). Screening of pesticide distributions in foods of animal origin: a matrix-based approach for biotransfer factor modeling of grazing mammals. *Environmental Science: Processes & Impacts*, *24*(4), 609–624. https://doi.org/10.1039/D1EM00454A

Longworth, K. E., Jones, J. H., Bicudo, J. E. P. W., Taylor, C. R., & Weibel, E. R. (1989). High rate of O2 consumption in exercising foxes: large Po2 difference drives diffusion across the lung. *Respiration Physiology*, *77*(3), 263–276. https://doi.org/10.1016/0034-5687(89)90115-1

Løvendahl, P., & Sehested, J. (2016). Short communication: Individual cow variation in urinary excretion of phosphorus. *Journal of Dairy Science*, *99*(6), 4580–4585. https://doi.org/10.3168/jds.2015-10338

Marsden, K. A., Lush, L., Holmberg, Jon. A., Whelan, M. J., King, A. J., Wilson, R. P., Charteris, A. F., Cardenas, L. M., Jones, D. L., & Chadwick, D. R. (2020). Sheep urination frequency, volume, N excretion and chemical composition: Implications for subsequent agricultural N losses. *Agriculture, Ecosystems & Environment*, *302*, 107073. https://doi.org/10.1016/j.agee.2020.107073

Mavroudis, P. D., Hermes, H. E., Teutonico, D., Preuss, T. G., & Schneckener, S. (2018). Development and validation of a physiology-based model for the prediction of pharmacokinetics/toxicokinetics in rabbits. *PLOS ONE*, *13*(3), e0194294. https://doi.org/10.1371/journal.pone.0194294

Morris, A. D., Muir, D. C. G., Solomon, K. R., Teixeira, C. F., Duric, M. D., & Wang, X. (2018). Bioaccumulation of Polybrominated Diphenyl Ethers and Alternative Halogenated Flame Retardants in a Vegetation–Caribou–Wolf Food Chain of the Canadian Arctic. *Environmental Science & Technology*, *52*(5), 3136–3145. https://doi.org/10.1021/acs.est.7b04890

Müller, C. E., De Silva, A. O., Small, J., Williamson, M., Wang, X., Morris, A., Katz, S., Gamberg, M., & Muir, D. C. G. (2011). Biomagnification of Perfluorinated Compounds in a Remote Terrestrial Food Chain: Lichen–Caribou–Wolf. *Environmental Science & Technology*, *45*(20), 8665–8673. https://doi.org/10.1021/es201353v

Murphy, M. R. (1992). Water Metabolism of Dairy Cattle. *Journal of Dairy Science*, *75*(1), 326–333. https://doi.org/10.3168/jds.S0022-0302(92)77768-6

My Food Data - Free Nutrition Tools to Understand What You Eat. (n.d.-a). Retrieved November 20, 2024, from https://www.myfooddata.com/

My Food Data - Free Nutrition Tools to Understand What You Eat. (n.d.-b). Retrieved January 2, 2025, from https://www.myfooddata.com/

Nutrition Facts Search Tool. (2025). https://tools.myfooddata.com/nutrition-facts

Paolo CAVALLINI. (1997). *Internal organ masses of the red fox Vulpes vulpes:data from the wild* . https://www.faunalia.eu/pdf/ActaTheriol97.pdf

Peng Yingjun. (1995). Preliminary Study on the Effect of Different Dietary Fat Levels on Bile Secretion in Bears with Artificial Bile Duct Drainage (Chinese). *Sichuan Animals*, *14*(1), 163–165.

Prince, H. (1982). BLOOD VOLUME IN THE PREGNANT RABBIT. *Quarterly Journal of Experimental Physiology*, *67*(1), 87–95. https://doi.org/10.1113/expphysiol.1982.sp002628

Reeves, P. T., Minchin, R. F., & Ilett, K. F. (1988). Measurement of organ blood flow in the rabbit. *Journal of Pharmacological Methods*, *20*(3), 187–196. https://doi.org/10.1016/0160-5402(88)90062-9

Reimers, E., Ringberg, T., & Sørumgård, R. (1982). Body composition of Svalbard reindeer. *Canadian Journal of Zoology*, *60*(8), 1812–1821. https://doi.org/10.1139/z82-235

Schladweiler, P., & Stevens, D. R. (1973). Weights of Moose in Montana. *Journal of Mammalogy*, *54*(3), 772. https://doi.org/10.2307/1378980

Siegal-Willott, J., Citino, S. B., Wade, S., Elder, L., Hayek, L.-A. C., & Lance, W. R. (2009). BUTORPHANOL, AZAPERONE, AND MEDETOMIDINE ANESTHESIA IN FREE-RANGING WHITE-TAILED DEER (ODOCOILEUS VIRGINIANUS) USING RADIOTRANSMITTER DARTS. *Journal of Wildlife Diseases*, *45*(2), 468–480. https://doi.org/10.7589/0090-3558-45.2.468

Smith, Y., H.O. de Waal, & O.B. Kok. (2006). Aspects of Carcass Digestibility by African Lions (Panthera leo Linnaeus, 1758) under Captive Conditions. *Pakistan Journal of Biological Sciences*, *9*(11), 2149–2152. https://doi.org/10.3923/pjbs.2006.2149.2152

Sutton, J. D. (1985). Digestion and Absorption of Energy Substrates in the Lactating Cow. *Journal of Dairy Science*, *68*(12), 3376–3393. https://doi.org/10.3168/jds.S0022-0302(85)81251-0

Symonds, H. W., Mather, D. L., & Hall, E. D. (1982). Surgical procedure for modifying the duodenum in cattle to measure bile flow and the diurnal variation in biliary manganese,

iron, copper and zinc excretion. *Research in Veterinary Science*, *32*(1), 6–11. https://doi.org/10.1016/S0034-5288(18)32428-7

Upton, R. N. (2008). Organ weights and blood flows of sheep and pig for physiological pharmacokinetic modelling. *Journal of Pharmacological and Toxicological Methods*, *58*(3), 198–205. https://doi.org/10.1016/j.vascn.2008.08.001

Valitskaya, R. I., Shishkin, G. S., & Voevoda, T. V. (1988). Ecological differences in structural organization of the respiratory regions of the lungs. *Bulletin of Experimental Biology and Medicine*, *106*(1), 1059–1061. https://doi.org/10.1007/BF00834679

Van Valkenburgh, B., Theodor, J., Friscia, A., Pollack, A., & Rowe, T. (2004). Respiratory turbinates of canids and felids: a quantitative comparison. *Journal of Zoology*, *264*(3), 281–293. https://doi.org/10.1017/S0952836904005771

Wei, Z., Wei Li, S., Lin Lin, M., Ke Yuan, L., & Fei Fei, H. (2020). Effects of Feeding Level on Productive Performance, Organ Development and Body Energy Deposition of Male Arctic Foxes during the Winter Fur Growth Period. *Journal of Veterinary Medicine*, *51*(1), 90–98.

WILLIAMS, P. (2007). Nutritional composition of red meat. *Nutrition & Dietetics*, *64*(s4). https://doi.org/10.1111/j.1747-0080.2007.00197.x

Williams, P. G. ,. (2007). *Nutritional composition of red meat*.

Xiao, X., Gao, Z., & Wang, L. (1998). HEMATOLOGICAL AND BLOOD BIOCHEMICAL REFERENCE VALUES OF WOLVES. *Acta Theriologica Sinica*, *1*, 34–41.

Yang, T. S., & Lin, J. H. (1997). Variation of heart size and its correlation with growth performance and vascular space in domestic pigs. *Animal Science*, *64*(3), 523–528. https://doi.org/10.1017/S1357729800016155

YUN Shi-feng, HU Yu hong, & TIAN Xiao yun. (2004, December). *Study on the Chief Organ Weight and Coefficients of Different Species of Experimental Rabbits*. https://www.doc88.com/p-0052021191593.html
